# microRNA-501 controls myogenin^+^/CD74^+^ myogenic progenitor cells during muscle regeneration

**DOI:** 10.1016/j.molmet.2023.101704

**Published:** 2023-03-11

**Authors:** Alexandra Fahrner, Edlira Luca, Jan Krützfeldt

**Affiliations:** 1 Division of Endocrinology, Diabetes, and Clinical Nutrition, University Hospital Zurich, 8091, Zurich, Switzerland; 2 Life Science Zurich Graduate School, Biomedicine, University of Zurich, 8057, Zurich, Switzerland

**Keywords:** microRNA, aging, Esrrg, CD74, skeletal muscle, regeneration, stem cells

## Abstract

**Objective:**

Skeletal muscle regeneration is markedly impaired during aging. How adult muscle stem cells contribute to this decrease in regenerative capacity is incompletely understood. We investigated mechanisms of age-related changes in myogenic progenitor cells using the tissue-specific microRNA 501.

**Methods:**

Young and old C57Bl/6 mice were used (3 months or 24 months of age, respectively) with or without global or tissue-specific genetic deletion of miR-501. Muscle regeneration was induced using intramuscular cardiotoxin injection or treadmill exercise and analysed using single cell and bulk RNA sequencing, qRT-PCR and immunofluorescence. Muscle fiber damage was assessed with Evan`s blue dye (EBD). *In vitro* analysis was performed in primary muscle cells obtained from mice and humans.

**Results:**

Single cell sequencing revealed myogenic progenitor cells in miR-501 knockout mice at day 6 after muscle injury that are characterized by high levels of myogenin and CD74. In control mice these cells were less in number and already downregulated after day 3 of muscle injury. Muscle from knockout mice had reduced myofiber size and reduced myofiber resilience to injury and exercise. miR-501 elicits this effect by regulating sarcomeric gene expression through its target gene estrogen-related receptor gamma (*Esrrg*). Importantly, in aged skeletal muscle where miR-501 was significantly downregulated and its target *Esrrg* significantly upregulated, the number of myog^+^/CD74^+^ cells during regeneration was upregulated to similar levels as observed in 501 knockout mice. Moreover, myog^+^/CD74^+^-aged skeletal muscle exhibited a similar decrease in the size of newly formed myofibers and increased number of necrotic myofibers after injury as observed in mice lacking miR-501.

**Conclusions:**

miR-501 and Esrrg are regulated in muscle with decreased regenerative capacity and loss of miR-501 is permissive to the appearance of CD74^+^ myogenic progenitors. Our data uncover a novel link between the metabolic transcription factor Esrrg and sarcomere formation and demonstrate that stem cell heterogeneity in skeletal muscle during aging is under miRNA control. Targeting Esrrg or myog^+^/CD74^+^ progenitor cells might improve fiber size and myofiber resilience to exercise in aged skeletal muscle.

## Highlights

• Genetic deletion of microRNA-501 reduces myofiber size and increases myofiber damage after injury and exercise
• miR-501 targets sarcomere formation through estrogen-related receptor gamma and controls myogenic progenitor cells that are characterized by the presence of myogenin and CD74
• Inhibition of miR-501 in human primary muscle cells recapitulates the changes in sarcomeric gene expression observed in miR-501 knockout mice
• miR-501 and its target gene Esrrg are regulated in aged muscle with decreased regenerative capacity
• myogenin^+^/CD74^+^ cells are upregulated during muscle regeneration in aged mice

## 1 Introduction

Recent advances in single cell sequencing technology have provided unparalleled accessibility to the various cells types found in the adult skeletal muscle in addition to those engaged during the process of muscle regeneration [[1], [2], [3], [4]]. Single cell sequencing has revealed 9-11 distinct cell types in adult skeletal muscle [[1], [2], [3]] and 15 distinct cell types during muscle regeneration [4], including 5-6 subpopulations of resident muscle stem cells (MuSCs) also called satellite cells [[4], [5]]. MuSCs express the transcription factor paired box-7 (Pax7) [6] and provide the skeletal muscle with a remarkable capacity to regenerate [7]. They are typically maintained in a quiescent state by external and internal molecular factors [7]. Upon injury, MuSCs are activated and re-enter the cell cycle. They vastly proliferate, expand into committed myogenic progenitor cells (MPs), and finally differentiate into newly formed myofibers. Pax7 and myogenic regulatory factors (MRFs) provide transcriptional control of muscle regeneration and serve as markers for the different subpopulations of satellite cells. *Pax7* identifies quiescent and activated stem cells. *Myf5* is the earliest marker of myogenic commitment among the MRFs and is already found in the majority of quiescent MuSCs [8]. Following activation, MuSCs upregulate *MyoD* expression, re-enter the cell cycle to proliferate and subsequently progress into differentiation [9]. Downregulation of *Pax7* and upregulation of *myogenin* (*Myog*) marks the differentiation process [9]. Throughout regeneration, a myriad of immune, endothelial, and fibroblastic cells create a microenvironment to promote myogenesis [10]. During the initial degenerative phase, damaged tissue attracts neutrophils, CD8^+^ cytotoxic T cells, and pro-inflammatory M1/LyC6^pos^ macrophages, promoting the proliferation of MuSCs. A subsequent transition to an anti-inflammatory phase characterised by regulatory T (T_reg_) cells and M2/LyC6^neg^ macrophages causes MuSCs to exit the cell cycle and differentiate. Furthermore, mesenchymal cells such as fibro/adipocyte progenitor cells (FAPs) and endothelial cells are responsible for the deposition of extracellular matrix and angiogenesis, respectively [[11], [12]].

Aging is characterized by a decline of the regenerative capacity of skeletal muscle [13]. This decline is attributed to a decrease in the pool size of satellite cells and their functional capacity to repair myofibers [[14], [15], [16], [17]]. These defects are caused by systemic factors that decline during aging [18] as well as by changes in the aged stem cell niche and intrinsic factors [[14], [19], [20], [21], [22]]. The role of microRNAs (miRNAs) in the functional decline of MuSCs during aging remains largely unexplored although miRNAs have emerged as promising biomarkers of the age-related loss of muscle mass [23] and are essential for every step of the regeneration process. MuSCs deficient of miRNAs exit quiescence [24] and fail to form myotubes [25]. Pharmacological inhibition in vivo using antagomirs showed that single miRNAs are involved in the maintenance of MuSC quiescence [[24], [26], [27]] or promotion of myoblast differentiation [[28], [29]]. However, genetic evidence for the impact of miRNAs on muscle regeneration is scarce. The global knockout of miR-206 delayed muscle regeneration, without affecting fiber size between knockout and control mice [30], while the MuSC-specific deletion of miR-29a decreased the proliferation rate of MPs and reduced muscle mass [31]. Whether miRNAs are involved in establishing stem cell heterogeneity during muscle regeneration is unknown.

In 2016, our group described a novel muscle-specific miRNA, miR-501-3p, enriched in activated MPs following cardiotoxin (CTX)-induced muscle regeneration [32]. miR-501 is located in a cluster of miRNAs within the second intron of the chloride channel 5 gene (Clcn5-2) on the X-chromosome. Silencing of miR-501 using antagomirs during muscle regeneration significantly decreased the diameter of newly formed myofibers [32]. Here, we expand on our observations of this miRNA and identify it as a critical determinant of stem cell heterogeneity during regeneration and predictor of myofiber size and resilience of mature myofibers in aged skeletal muscle.

## 2 Material and Methods

### 2.1 Human biopsy acquisition

Human myogenic progenitors were isolated from biopsies from the Tensor Fasciae Latae muscle obtained during elective hip replacement surgery as previously described (2 females and 2 males, age 40.5 ± 6.3) [33].

### 2.2 Mice

Unless otherwise indicated, all animals used were 12-week-old male mice. Aged miR-501^ΔMP^ mice and control littermates were assessed at 24 months of age. All animals housed at 2-5 littermates per cage in individually ventilated cages under conditions of controlled temperature (22 °C) and illumination (12-h light/12-h dark cycle; light off at 6 p.m.) with *ad libitum* access to chow and water. All experiments were approved by the Veterinary office of the Canton of Zurich (License number 061/2019) and health status of all mouse lines were monitored on a regular basis according to FELASA guidelines. To induce muscle regeneration, tibialis anterior muscles were injected with Cardiotoxin (CTX, Sigma, 50 μl, 10 μM in PBS) or glycerol (Sigma, 50 μl, 50:50 v/v in PBS) under anesthesia as previously described [32]. Gene expression analysis in skeletal muscle from aged mice (22 months) was performed in a cohort previously described [25].

### 2.3 Generation of miR-501 knockout mouse models

miR-501^flox/flox^ mice have been generated by introducing loxP sites surrounding the miR-501 precursor (Cyagen, Santa Clara, US). C57BL/6 mouse genomic fragments containing homology arms (HAs) and a conditional knockout (cKO) region were amplified from BAC clones using high fidelity Taq polymerase and were sequentially assembled into a targeting vector together with recombination sites and selection markers. The vector was delivered to ES cells (C57BL/6) via electroporation and subsequently F1 mating with C57BL/6 mice was set up. B6.C-Tg(CMV-Cre)1Cgn/J mice (Jackson; No. 006054[34]) were crossed with miR-501^flox^ mice to achieve a hemizygous F1 generation that was backcrossed to generate a global deletion of miR-501 (miR-501^gKO^) and eliminate CMV^Cre^ expression. Non-inducible Pax7^tm1(Cre)Mrc/J^ mice (Jackson; No. 010530[35]) were purchased at the Jackson Laboratory and crossed with miR-501^flox^ mice to achieve excision of miR-501 specifically in the myogenic lineage (Pax7^Cre/+^miR-501^fl/Y^; miR-501^ΔMP^). Primer sequences to determine genotypes are listed below.

### 2.4 Blood Parameters

Blood glucose was measured in blood from tail vein using a glucometer (FreeStyle Lite, Abbott) after food starvation for 5 h. Intraperitoneal glucose tolerance test (IPGTT) was performed by injecting a 20 % glucose solution (Bichsel) at 10 μl per gram body weight after overnight food deprivation and measuring blood glucose in blood from tail vein at baseline (before injection), and in intervals of 15 min after injection up to 120 min. Serum parameters were measured in serum from heart blood mice directly after sacrification. For total triglyceride and cholesterol levels, the Colorimetric Serum Triglyceride Quantification Kit and the Colorimetric Total Cholesterol Assay Kit from Cell Biolabs was used respectively. To assess serum creatine kinase, the Creatine Kinase Activity Assay Kit was purchased from Sigma. All assays were performed following manufacturer’s instructions.

### 2.5 Eccentric Exercise

Mice were exercised on a five-lane treadmill (Panlab, Harvard Bioscience) for 90 min and an initial speed of 5 cm/s. The speed was gradually increased at increments of 1 cm/s every 30 s until a final speed of 25 cm/s was reached and the slope was decreased to -20° until the end of the exercise.

### 2.6 Grip Strength measurement

Grip strength of all four limbs was assessed using a grip strength meter (Bioseb). Mice were placed on a metal grid attached to the device and lifted by the tail. The maximal grip strength was measured in grams and an average of three consecutive measurements was recorded.

### 2.7 Evans Blue Dye

Evans Blue Dye (Sigma, 10 mg / ml in PBS) was injected intraperitoneally (0.1 ml per 10 g body weight) 18 h before scarification. The presence of EBD in frozen sections was detected as red auto-fluorescence.

### 2.8 Cell preparation and FACS

Procedures for isolation of primary muscle cells from human and mouse skeletal muscle were performed as previously described [31]. Mouse skeletal muscle tissue was excised from the hind limbs. Muscles were minced and digested with 2 mg/mL collagenase type II (Gibco) in collagenase buffer (1.5% BSA in HBSS) for 1 h at 37 °C. Muscle slurries were filtered through a 100 μm cell strainer (BD Biosciences), followed by treatment with erythrocyte lysis buffer (154 mM NH_4_Cl, 10 mM KHCO_3_, 0.1 mM EDTA) and filtration using 40 μm cell strainers (BD Biosciences). Cells were resuspended in washing buffer consisting of PBS with 0.5% BSA and stained with antibodies for 1 h at 4°C. Cell sorting was performed on a FACSAria III 4L (BD Biosciences). Mouse myogenic progenitors were isolated from live cell population (7-AAD^–^; Sigma) based on positive staining for α7-integrin (R&D, FAB3518P) and absence of Sca1 (BioLegend, 108111), CD31 (BioLegend, 102414) and CD45 CD45 (BioLegend, 103121) staining, while fibro/adipogenic progenitors were sorted based on positive staining for Sca1 and absence of staining for α7-integrin, CD31 and CD45. Human myogenic progenitors were sorted by excluding CD31 (BioLegend, 303103) / CD45 (BioLegend, 304019) and based on positive CD56 (BioLegend, 319305) staining and absence of CD15 (BioLegend, 323007).

For isolation of stromal vascular fraction (SVF), adipose tissue from subcutaneous (inguinal), visceral (perigonadal), and brown (interscapular) fat pads were dissected, minced and digested in collagenase buffer, filtered and subsequently plated.

### 2.9 Cell culture

Primary human and mouse myoblasts were cultured on collagen-coated plates in 1:1 v/v DMEM and Ham's F-10 Nutrient Mix (Gibco) containing 20% FBS, 1% P/S and 5 ng/mL recombinant human FGF-2 (Gibco). Differentiation was initiated when myoblasts reached subconfluency by changing the media to DMEM containing 2% horse serum (Gibco) and 1% P/S. Experiments with myotubes were initiated after 6 days under low-serum conditions, when myoblasts were fully differentiated. FAPs were cultured in DMEM supplemented with 20 % FBS, 1 % P/S, and 5 ng / mL FGF-2. SVF was cultured in DMEM supplemented with 10 % FBS and 1 % P/S on collagen-coated plates. All cell cultures were incubated in a 37 °C 5% CO_2_ water-jacketed incubator. For miR-501 inhibition, cells were transfected with 12 nM antagomirs or respective control antagomirs. Antagomirs were designed against miR-501-3p (CCAAAUCCUUGCCCGGGUGCAUU) or a scrambled control (ACACACAACACUGUCACAUUCCA) and custom synthesized by Sigma using modifications of a cholesterol molecule linked to the 3`end, complete 2`O-methylation and phosphorothioate linkages at the first two and the last four nucleotides as previously described [36]. Transfections of oligonucleotides were performed using Lipofectamine RNAiMAX (Invitrogen) according to the manufacturer`s protocol. For RNAi, 2 μg / mL of esiRNA (Sigma) targeting mouse Esrrg or EGFP (Control) were used. For Esrrg overexpression, 500 ng / mL of pcDNA3-Esrrg or pcDNA3 vectors (described in [32]) were transfected using Lipofectamine 2000 (Invitrogen).

### 2.10 RNA extraction, cDNA synthesis, quantitative RT-PCR

Total RNA was isolated using TRIzol Reagent (Invitrogen) according to the manufacturer's instruction. Traces of genomic DNA were removed using the DNA-free DNA Removal Kit (Invitrogen). Equal amounts of RNA were reverse-transcribed with random hexamer primers using SuperScript III First-Strand Synthesis System (Invitrogen). For miRNA qRT-PCR, 10 ng of total RNA was reverse-transcribed using the TaqMan MicroRNA Reverse Transcription Kit (Applied Biosystems). Quantitative RT-PCR for miRNA and mRNA levels were performed on a Quant Studio 5 Real-time PCR system (Applied Biosystems) using TaqMan Fast Universal PCR Master Mix, no AmpErase UNG (Applied Biosystems) and PowerUp SYBR Green Master Mix (Applied Biosystems), respectively. Gene expression was calculated using the Relative Standard Curve Method and 18S rRNA, as indicated, for normalization. All primer sequences are given in Table S1. The levels of miRNA were calculated using the ΔΔCt method and snoRNA234 or U6 snRNA for normalization. TaqMan assays for miR-501 (human: TM 002435, mouse: TM001651), miR-362 (TM: 002614), snoRNA234 (TM: 001234), and U6 snRNA (TM: 001973) were purchased from Applied Biosystems.

### 2.11 LDH Activity

miR-501^ΔMP^ myofibers were treated with CTX for 1 hour at the final concentration of 0.2 μM. LDH release was assessed using the CyQUANT LDH Cytotoxicity Assay (Invitrogen). 50 μl of cell culture media were incubated with 50 μl of Reaction Mixture for 30 min at room temperature, followed by the addition of 50 μl of Stop Solution. Absorbance was measured at 490 nm and background values of 680 nm were subtracted. Data were normalized to total protein content assessed using the Pierce BCA Protein Assay Kit (Thermo Sientific) and expressed as fold change vs control.

### 2.12 Proliferation assay

Proliferation rates in miR-501^ΔMP^ myoblasts were measured using Click-iT EdU Alexa Fluor 488 Flow Cytometry Assay Kit (Invitrogen) according to manufacturer's instruction. Briefly, transfected cells were treated with 5 μM EdU for 6 h. Cells were trypsinized and washed with 1% BSA in PBS. Cells were then fixed, permeabilized, and stained with the click reaction using Alexa Fluor 488 azide. Flow cytometry was performed on the LSR II Fortessa cell analyzer (BD Biosciences). Analysis and determining the percentage of EdU-positive cells was performed using FlowJo software (v10.6.2, BD Biosciences).

### 2.13 Fusion Index and fiber diameter

miR-501^ΔMP^ myofibers were fixed with 4% PFA and stained with Wheat Germ Agglutinin (WGA, Alexa Fluor 594, Invitrogen, 5 μg/mL in PBS) followed by blocking and permeabilising (0.5% Triton-X 100 in PBS) and staining with DAPI (Invitrogen, 1:1000) for 15 min at room temperature each. Cells were imaged using a Cytation 5 Cell Imaging Multi Mode Reader (Biotek). Fusion index was calculated as the ratio of nuclei inside a myotube containing at least three nuclei compared to the number of total nuclei in the image after manual counting using the Cell Counting tool, fiber diameter was measured as an average of three measurements in fibers with more than 3 nuclei per image in ImageJ (v. 1.53c).

### 2.14 Protein extraction and western blot

Cells and skeletal muscle samples were lysed in RIPA Buffer (50 mM Tris-HCl pH 7.4, 150 mM NaCl, 2mM EDTA, 1 % Triton X100, 0.5 % sodium deoxycholate) supplemented with protease (Complete, Roche) and phosphatase (PhosSTOP, Roche) inhibitor cocktails. Lysates were cleared by centrifugation at 14,000 × g for 20 min at 4 °C. Protein concentrations were determined using BCA Assay. Equal amounts of protein (20 μg) were separated by SDS-PAGE, transferred onto Protran Nitrocellulose Membranes (GE Healthcare) using eBlot L1 Fast Wet Transfer System (GenScript), followed by incubation with indicated primary antibodies against α-actinin (R&D, MAB9830; 1:1000), LC3 (Abcam, ab192890; 1:1000), MuRF1 (Santa Cruz, sc-398608; 1:1000), MuRF2 (Abcam, ab4387; 1:1000), and γ-tubulin (Sigma-Aldrich T-5326; 1:1000). Signals of anti-rabbit IgG horseradish peroxidase-conjugated secondary antibody (1:10’000) were visualized on a LAS-3000 Luminescent Image Analyzer (Fujifilm) using Lumi-Light Western Blotting Substrate (Roche).

### 2.15 Histology

Mouse Tibialis anterior muscles were fixed in 4% formalin and embedded in paraffin using the Excelsior AS Tissue Processor (Thermo Scientific). Sections of 4 μm thickness were prepared using a fully automated rotary microtome (RM2255, Leica Biosystems). Sections were de-paraffinised and rehydrated, stained with hematoxylin (Harris) and eosin (Morphisto) or subjected to immunofluorescence using antigen retrieval with citrate buffer (0.01 M trisodium citrate dihydrate in H2O). After blocking (10 % FBS, 1 % Triton-X 100 (Sigma) in PBS), primary antibodies against CD138 (BioLegend, 142501; 1:400) and IgA (GeneTex, GTX77225; 1:400) were incubated overnight at 4°C. Myogenin (Santa Cruz, sc-12732; 1:100) and CD74 (BioLegend, 151002; 1:200) staining was performed using the Mouse on Mouse Immunodetection Fluorescein Kit (Vector Laboratories). The next day, Alexa Fluor 488 and 594-conjugated secondary antibodies (Invitrogen, 1:500), and DAPI (Invitrogen, 1:1000) were used for 1 h at room temperature.

For frozen sections, mouse tibialis anterior muscles were snap frozen in isopentane/liquid nitrogen and cut at 10 μm thickness on a cryostat (Leica CM1860). Staining for laminin (Sigma L9393; 1:500) and eMHC (DSHB, BF-G6; 1:20) using antigen retrieval with citrate buffer and the Mouse on Mouse Kit was performed as previously described[32]. Pax7 (DSHB; 1:20) as well as Pax7-CD74 (BioLegend, 151002; 1:200) co-staining was performed analogously. Muscle fiber staining of MHC I (DSHB, BA-F8; 1:20), MHC IIa (DSHB, Sc-71; 1:20), and MHC IIb (DSHB, BF-F3; 1:20) was performed using a similar protocol, omitting fixation with PFA and antigen retrieval. All immunofluorescence analyses were done per whole muscle cross section. All sections were scanned using an Axio Scan.Z1 slide scanner (Zeiss) equipped with ZEN Imaging software v3.1 and analysed using ImageJ (v. 1.53c). ROI colour coder plugin for ImageJ was used to show size distribution of muscle fibers.

### 2.16 Sarcomere analysis

For sarcomere staining *in vitro*, myotubes were differentiated on Nunc Lab-Tek Chamber Slides (Thermo Scientific) and fixed in 4% paraformaldehyde at day 6 of differentiation. Staining was performed by blocking slides with 0.5% Triton-X 100 in PBS, and incubating α-actinin antibody (R&D, MAB9830; 1:400) overnight at 4 °C. The next day, fibers were incubated with secondary antibody (1:500) and DAPI (1:1000) at room temperature for one hour. Fibers were imaged using a Leica SP8 inverted confocal laser scanning microscope and sarcomere length was assessed using the Find Peak plugin for ImageJ on the staining histogram.

### 2.17 Single cell RNA-sequencing

For single-cell experiments, TA muscles of 3 Pax7^Cre^ and 3 miR-501^ΔMP^ mice were pooled, and sequencing was performed in two independent experiments. Freshly FACS-sorted myogenic progenitors (CD45^-^ / CD31^-^ / Sca1^-^ / α7integrin^+^) were used to acquire 10X-based libraries using the Chromium Single cell V3.0 reagent kit (10X Chromium) according to manufacturer’s instruction. Cell suspensions of roughly 500 cells per μl was loaded and sequenced using a NovaSeq 600 (Illumina). Data was analysed using the mkfast and count pipeline in Cellranger (v2.0.2) Gene list for GO muscle contraction (GO:0006936) was obtained from the Mouse Genome Informatics database [37].

### 2.18 RNA-seq

After RNA isolation, RNA quality control was performed on the 4200 TapeStation system (Agilent). Sequencing libraries were prepared from 500 ng total RNA using the TruSeq Stranded mRNA Library Prep kit (Illumina) according to manufacturer's instruction. Illumina flow cells were prepared, and samples sequenced on an Illumina NovaSeq 6000 instrument to generate 100 bp single-end reads. RNA-seq reads were aligned with STAR-aligner using the gene annotation as provided by ENCODE release 91. Gene ontology analysis was performed using Enrichr [[38], [39]].

### 2.19 Statistical analysis

Numerical values are reported as mean ± SEM. Sample sizes were determined on the basis of previous experiments and publications using similar methodologies as well as on observed effect sizes. For *in vivo* studies, animal numbers are indicated in the figure legends for all experiments. Cell culture experiments were performed with two technical replicates and reproduced using independent cell cultures. Numerical values are shown as mean ± SEM. Statistical significance (∗*p* ≤ 0.05; ∗∗*p* ≤ 0.01; ∗∗∗*p* ≤ 0.001) was evaluated as specified in the figure legends using GraphPad Prism v9.1.0.

## 3 Results

### 3.1 Deletion of miR-501 reveals unique myogenic progenitor cells enriched for macrophage markers

**Figure 1:**
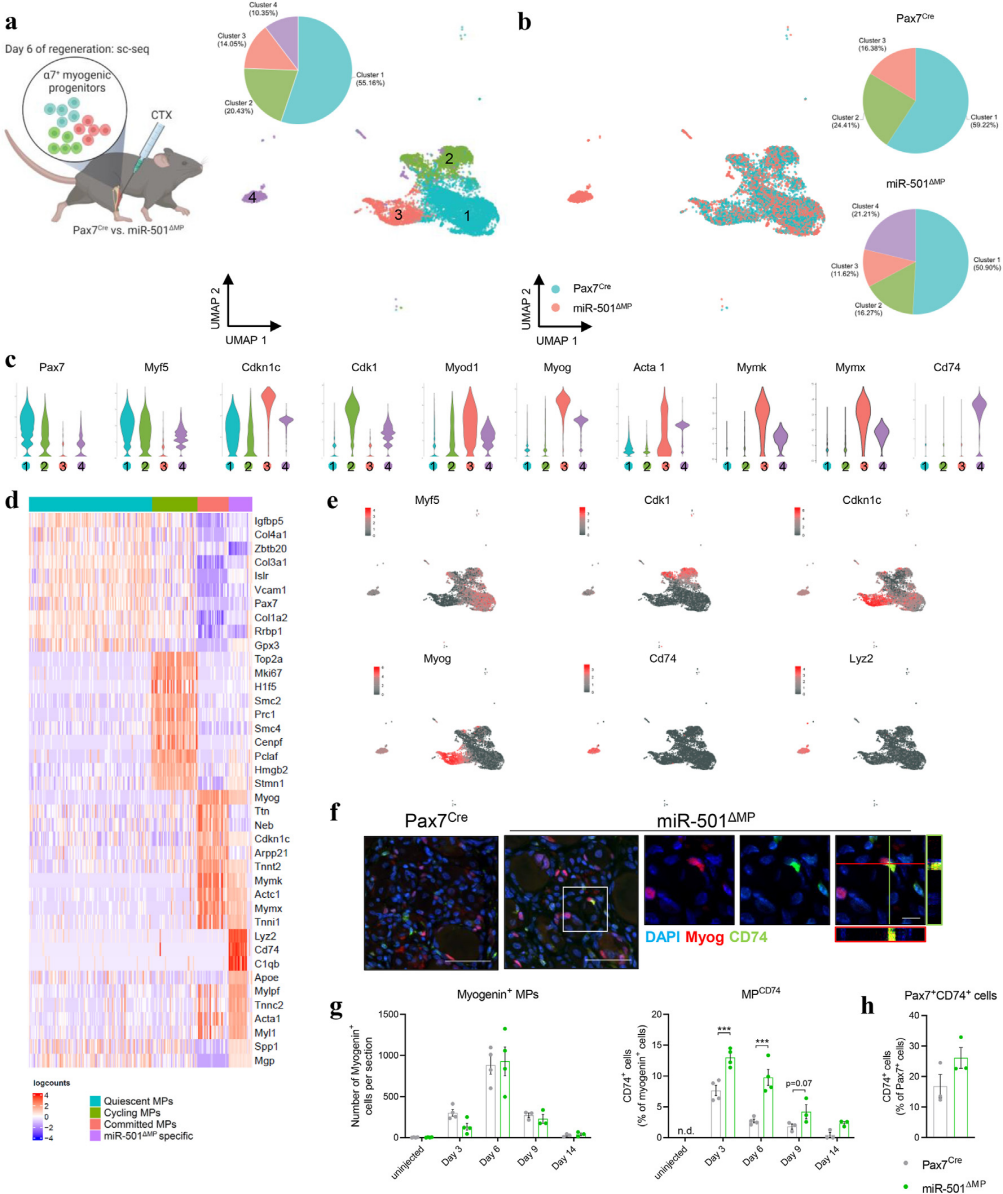
**scRNA-seq reveals a novel MP subpopulation in skeletal muscle lacking miR-501. a** Graphical abstract of experimental setup at day 6 of CTX-induced regeneration and integrated analysis of snRNA-seq including 8055 myogenic progenitor cells (MPs) merged from two independent experiments. Visualisation of MP sub-populations and cell count is shown colour coded for clusters 1-4. **b** Cluster composition by condition: Pax7^Cre^ (4122 cells) and miR-501^ΔMP^ (3933 cells). **c** Violin plot of scRNA expression profiles of marker genes for each cluster. **d** Heatmap of top 10 marker genes for quiescent stem cells (Qui), cycling stem cells (Cy), committed progenitors (Com), and novel miR-501^ΔMP^ specific cluster. **e** Feature plots of *Myf5* (Qui), *Cdk1* (Cy), *Cdkn1c* and *Myog* (Com), *Cd74* and *Lyz2* (miR-501^ΔMP^ specific) **f** Immunofluorescence of Myogenin, CD74, and DAPI in transverse muscle sections at day 6 of CTX-induced regeneration; scale bar = 50 μm (left), 10 μm (right, cutout). **g** Quantification of Myogenin^+^ cells and MP^CD74^ (Myogenin^+^CD74^+^) cells per section at different days following CTX-induced muscle injury. **h** Quantification of Pax7^+^CD74^+^ per section at day 6 following CTX-induced muscle injury; n = 3. Experiments were performed on 12-week-old male mice and all data is shown as mean ± SEM. d shows representative images and g the analysis of two sections at two different heights within the muscle. Significance was evaluated by two-way analysis of variance (ANOVA); ∗∗∗*p* ≤ 0.001.

Muscle fibers arise from a heterogeneous pool of adult muscle stem cells (MuSCs) [[1], [2], [3], [4], [5]] and during regeneration the various subpopulations of MuSCs can be found in a quiescent, cycling or committed state. We reasoned that the effect of miR-501 on the diameter of new myofibers might arise from changes in the ratios of these three states during regeneration. Therefore, we used single cell sequencing in a genetic mouse model for miR-501 to understand how miR-501 affects MuSC heterogeneity. We generated a novel genetic model (described in detail in the next section) where MuSC-specific deletion of miR-501 (miR-501^ΔMP^) was achieved using the paired box 7 promoter (Pax7^Cre^), successfully eliminating the miRNA from MuSCs in skeletal muscle. The targeting vector was designed to delete the precursor for miR-501-3p and miR-501-5p. Since our RNA sequencing results previously revealed that miR-501-5p is not expressed in activated MPs (<0.005 % of all miRNA sequences versus 5.3% for miR-501-3p [32]) we refer in the following to miR-501-3p. We combined this genetic model with the cardiotoxin (CTX) model of muscle injury to induce regeneration in the adult skeletal muscle and employed single cell sequencing on FACS-sorted MuSCs 6 days after intramuscular injection of CTX in the tibialis anterior (TA) muscle of Pax7^Cre^ and miR-501^ΔMP^ mice. We removed cells with fewer than 5000 unique molecular identifiers (UMIs) or 1000 detected genes and merged the two datasets, resulting in the profiling of 8055 high-quality cells (Fig. S1a). Unsupervised clustering analysis of the single cell sequencing dataset identified 4 distinct MuSC subpopulations (Fig. 1a,b). Intriguingly, cluster 1, 2, and 3 contained cells from both control and miR-501^ΔMP^ mice, whereas cluster 4 was exclusively detected in miR-501^ΔMP^, comprising 21% of total MuSC cells (834 out of 3933; Fig. 1b, Fig. S1b,c). Based on the presence of *Pax7* and *Myf5* and absence of *Myod1* (cluster 1), the presence of *Myod1* and the cell cycle gene *Cdk1* (cluster 2) and the presence of *Myog* and markers for myogenic differentiation/inhibition of cell cycle (*Acta1*, *Cdkn1c*) (cluster 3), cluster 1, 2 and 3 could be assigned to quiescent, cycling and committed MuSCs, respectively (Fig. 1c). High expression levels of terminal differentiation markers (*Myog, Acta1, Cdkn1c, Tnnt2, Myl1*) in the miR-501^ΔMP^-specific cluster 4 indicated that this cluster is closely related to committed progenitors (Fig. 1d). In addition, cluster 4 revealed also high expression of the two muscle genes myomaker (*Mymk*) and myomixer (*Mymx*) that are essential for the fusion of myoblasts with injured myofibers [40] (Fig.1d). Top 10-marker gene analysis revealed a key characteristic of the miR-501^ΔMP^-specific cell cluster, a macrophage-specific gene signature (*Lyz2*, *Cd74*, and *C1qc*, Fig. 1c-e). Given the similarities with committed cells, we subsequently refer to this cell cluster as MP^CD74^.

To exclude batch effects, the single cell sequencing dataset came from two independent experiments performed on a pool of 3 mice per group (total of 6 mice per genotype). Side by side comparison of the two independently performed sequencing experiments revealed heterogeneity within the MP^CD74^ cells. MP^CD74^ in run 1 was homogenous in its expression of differentiation markers, while MP^CD74^ in run 2 was more heterogeneous and expressed both quiescent and committed markers (Fig. S1d). Slingshot analysis confirmed the trajectory of MuSCs as a branched progression from quiescent to cycling and committed progenitors [4] (Fig. S1e). MP^CD74^ was annotated in run 1 as fully progressed through the pseudo-timeline in a trajectory independent of the trajectory of committed progenitors, whereas in run 2 MP^CD74^ fell within the trajectory of commitment (Fig. S1e). The differences between run 1 and run 2 likely reflect different snap shots of MuSCs progressing through muscle regeneration. Immunofluorescent analysis for cells double-stained for myogenin and CD74 confirmed the presence of this unique cell cluster in miR-501^ΔMP^ mice, but not control littermates (Fig. 1f). Semi-automatic immunofluorescence analysis of full muscle sections showed no difference in the absolute number of *Myog*^*+*^ cells between miR-501^ΔMP^ mice and controls (Fig.1g). MP^CD74^ are not detected in uninjected muscles. In control mice, MP^CD74^ are detectable at day 3 followed by a rapid downregulation. Less than 5 % of *Myog*^*+*^ cells are positive for CD74 in control mice at day 6 after muscle regeneration. In contrast, miR-501^ΔMP^ mice show already a more pronounced induction of MP^CD74^ at day 3 compared to control mice and a slower decline with over 10 % of *Myog*^*+*^ cells being also CD74^+^ (Fig.1g). There was no significant difference for Pax7^+^/CD74^+^ cells between wildtype and knockout mice (Fig.1h). However, we were not able to combine myogenin staining with Pax7 and CD74 and it is therefore not clear to what extend myogenin^+^/CD74^+^ and Pax7^+^/CD74^+^ cells overlap. Taken together, we conclude that loss of miR-501 alters the heterogeneity of MuSCs, resulting in the emergence of a lateral lineage of committed MPs.

### 3.2 miR-501 determines myofiber size in adult skeletal muscle

**Figure 2:**
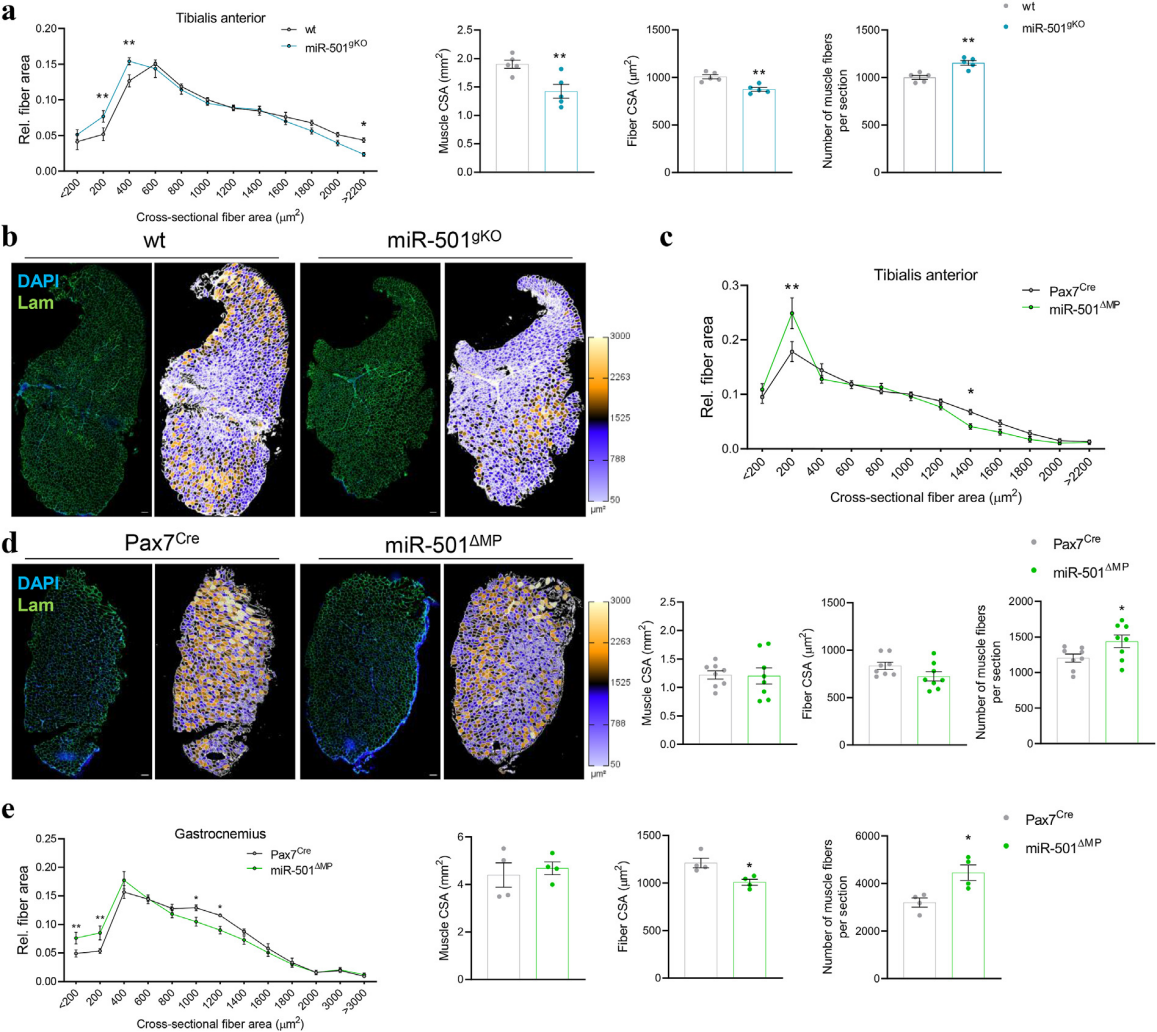
**Deletion of miR-501 leads to reduced muscle fiber diameter. a - b** Muscle cross sections of wt and miR-501^gKO^ mice were subjected to anti-laminin immunofluorescence and quantified for the distribution of muscle fiber size, cross-sectional area (CSA) muscle sections and fibers, and fiber number each section (**a**); *n* = 5. **b** representative images and colour coding of fiber size. **c - e** Muscle cross sections of Pax7^Cre^ and miR-501^ΔMP^ mice were assessed for the distribution of fiber size in TA muscle (**c**; *n* = 8) as well as Gastrocnemius (Gas; **e**, *n* = 4) based on anti-Laminin immunofluorescence. Representative images and colour coding of TA staining is shown in **d**. Muscle CSA, average fiber size and fiber number are shown for TA (**c**; *n* = 8) and Gas (**e**; *n* = 4). Experiments were performed on 12-week-old male mice and all data is shown as mean ± SEM. a, c, and e represent the analysis of 2 - 5 sections at different height per mouse; Scale bar = 100 μm. Significance was evaluated by t-test (a, d, e), and two-way analysis of variance (ANOVA; a, c, e); ∗: p < 0.05, ∗∗: p < 0.01.

To understand how the MP^CD74^ cells influences muscle structure and function, we used two novel knockout mouse models using flox sites surrounding the miR-501 precursor, generating a global genetic deletion and the MP-specific genetic deletion of miR-501 mentioned previously (Fig. S2a). miR-501, pri-miR-501 and the host gene *Clcn5-2* are enriched in MPs compared to uninjured muscle tissue and other precursor cell types (Fig. S3a). Global deletion of miR-501 (miR-501^gKO^) using the human cytomegalovirus minimal promoter (CMV^Cre^; Fig. S1b) efficiently depleted miR-501 in all cell types (Fig. S3b). miR-501^gKO^ mice had no alterations in body weight, muscle weight and strength, and glucose and lipid metabolism (Fig. S3c). Deletion of miR-501 did not alter the number of Pax7^+^ MuSCs (Fig. S3d). We did not observe changes in the formation of intramuscular adipose tissue (IMAT) in miR-501^gKO^ mice after muscle injury using the glycerol model, suggesting that the miRNA is not involved in other aspects of muscle regeneration (Fig. S3e). However, tibialis anterior (TA) muscles from miR-501^gKO^ mice showed a shift towards smaller diameter muscle fibers (Fig. 2a,b), decreased average muscle and muscle fiber cross-sectional area (CSA), alongside a significant increase in the number of total fibers per muscle section (Fig. 2a). A similar shift towards smaller fiber diameters was observed when the glycerol model of muscle injury was used (Fig. S3e). The results from the global deletion of miR-501 are in line with our previously reported role of miR-501 on myofiber size using pharmacological inhibition of the miRNA [32].

MP-specific deletion of miR-501 (miR-501^ΔMP^) using the paired box 7 promoter (Pax7^Cre^; Fig. S2c) largely mirrored the findings from the miR-501^gKO^ mice. Myofiber diameter was once again shifted to smaller sized fibers in TA as well as in gastrocnemius muscles (Fig. 2c,d,e), while fiber number was significantly increased (Fig. 2c,e). Body weight, muscle weight and strength, and glucose and lipid metabolism were unchanged in miR-501^ΔMP^ mice (Fig. S4a,b). Additionally, we did not find evidence for muscle damage/fiber regeneration in the non-stressed basal state (absence of embryonic myosin heavy chain (eMHC^+^) fibers, Fig. S4c) or altered fiber typing (Fig. S4d). Lastly, the proteasome and autophagy pathways were unaffected in the basal state (Fig. S4e). Together, these results indicate that miR-501 acts upon MPs to determine myofiber size in two independent muscle groups. Loss of miR-501 is permissive to the appearance of the tangential MP^CD74^ cells, which perturbs myofiber size as newly formed fibers commit and mature postnatally.

### 3.3 miR-501 is necessary for neofiber formation and resilience in the adult skeletal muscle

**Figure 3:**
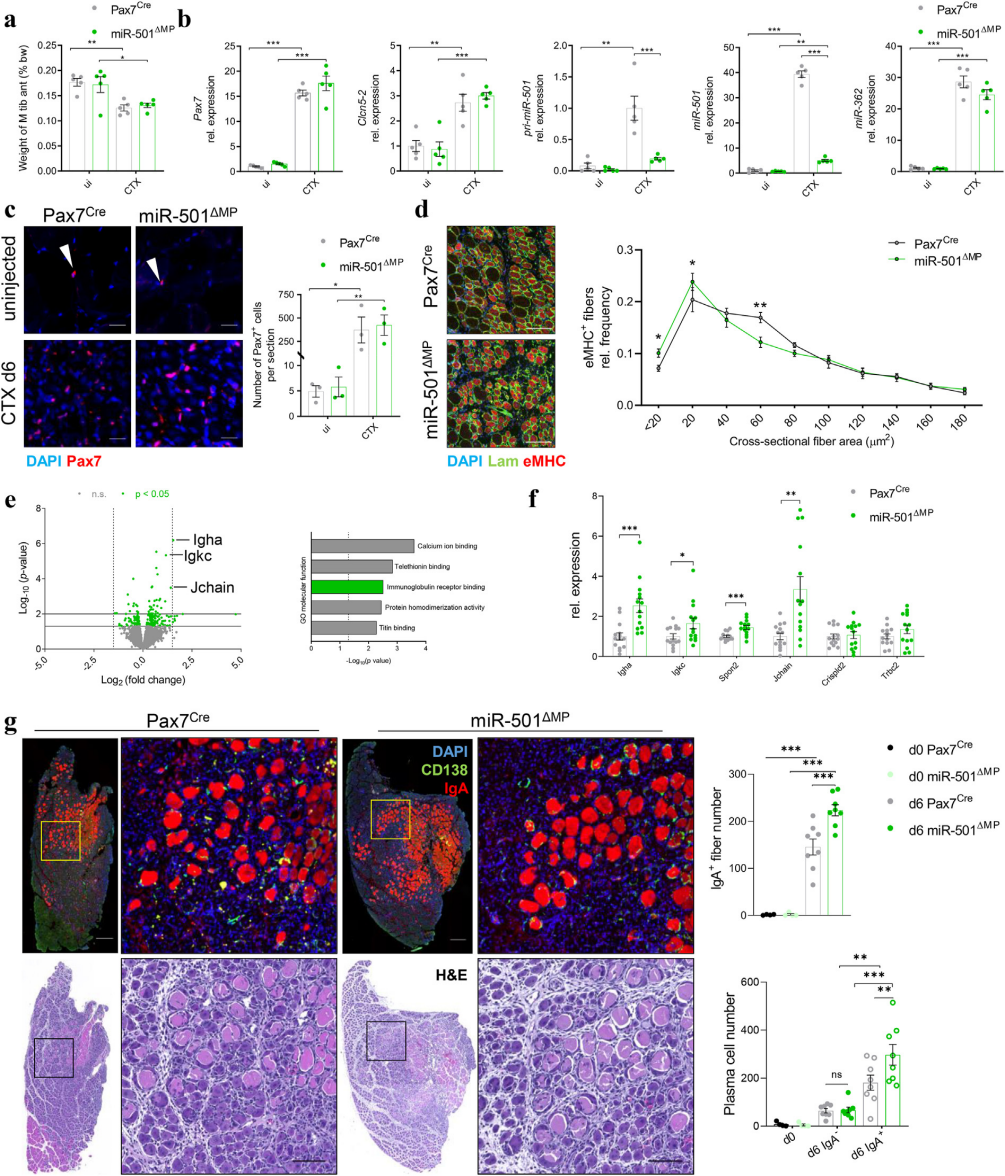
**Deletion of miR-501 results in smaller neofiber diameter and increased inflammatory response to damaged fibers during muscle regeneration. a** CTX was injected in TA muscles of Pax7^Cre^ and miR-501^ΔMP^ mice and TA muscle weight was measured as percent of total body weight in the uninjected muscle as well at day 6 of regeneration; *n* = 5. **b** Expression of *Pax7*, *Clcn5-2*, pri-miR-501, miR-501, and miR-362 in TA muscle at day 6 of regeneration compared to uninjected muscle; *n* = 5. **c** Muscle cross sections of uninjected or regenerating (day 6) TA muscle were stained using anti-Pax7 and DAPI immunofluorescence. Sections were quantified based on Pax7^+^ cells per section; *n* = 3. **d** Muscle cross sections of TA muscles at day 6 were stained using anti-Laminin, anti-eMHC, and DAPI immunofluorescence and shown as size distribution of the cross-sectional fiber area ; *n* = 3. **e** Volcano plot of RNA-seq analysis showing differential gene expression in TA of Pax7^Cre^ and miR-501^ΔMP^ muscles 6 days after CTX injury; *n* = 5, and Gene ontology (GO) pathway analysis for molecular function. **f** Expression of immune signature genes in TA at day 6; *n* = 14. **g** Sequential muscle cross sections of were stained using anti-CD138, anti-IgA, and DAPI immunofluorescence as well as hematoxylin and eosin (H&E) staining and quantified for IgA^+^ fiber number and plasma cell distribution within the regenerating muscle areas ; *n* = 4 (d0), *n* = 8 (d6). Experiments were performed on 12-week-old male mice and all data is shown as mean ± SEM; qPCR data was normalized to snoRNA234 (mmu-miR-501, miR-362) or 18S ribosomal RNA. c, d, and g show representative images and represent the analysis of two consecutive sections at two different heights within the muscle per mouse. Scale bar: 20 μm (Pax7), 100 μm (eMHC; H&E), 200 μm (IgA). Significance was evaluated by t-test (f), one-way- (a, b, c, g) and two-way analysis of variance (ANOVA; d); ∗: p < 0.05, ∗∗: p < 0.01, ∗∗∗: p < 0.001.

**Figure 4:**
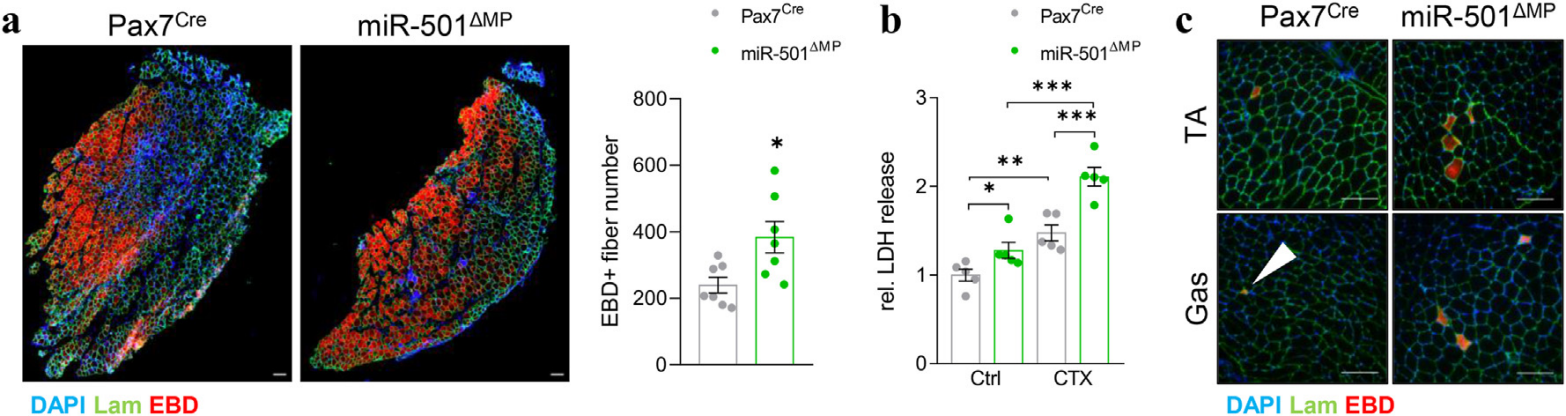
**Loss of miR-501 in myofibers increases susceptibility to damage during injury and exercise. a** Necrosis of myofibers at day 2 of CTX-induced muscle regeneration as determined by EBD^+^ fibers; *n* = 7. **b** Damage induced by CTX treatment determined by LDH release into *in vitro* culture media. Mouse primary myoblasts isolated from Pax7^Cre^ and miR-501^ΔMP^ mice were differentiated into myotubes for 6 days prior to CTX treatment; *n* = 5 independent cell cultures. **c** Damaged muscle fibers as a result of eccentric exercise, quantified in Table 1. Experiments were performed on 12-week-old male mice and all data is shown as mean ± SEM. a and b show representative images; b represents the analysis of 4 sections at different heights within the muscle per mouse. Lam = Laminin. Scale bar = 100 μm. Significance was evaluated by t-test (a) and one-way analysis of variance (ANOVA; b); ∗: p < 0.05, ∗∗: p < 0.01, ∗∗∗: p < 0.001.

To investigate how the expression of miR-501 in MPs impacts myofiber formation, we took an unbiased approach and performed bulk RNAseq analysis following CTX-induced injury to the TA muscle of miR-501^ΔMP^ and control littermates. 6 days after intramuscular CTX-injection, muscle weight and the expression of the MP marker Pax7 was unchanged in miR-501^ΔMP^ mice despite the depletion of miR-501 and its precursor (Fig. 3a,b). Notably, the expression of the host, *Clcn5-2*, as well as its closest neighbouring miRNA, miR-362 [32], were not affected by the deletion of the miR-501 gene (Fig. 3b). MP-specific deletion of miR-501 did also not alter the number of Pax7^+^ MuSCs in non-regenerating muscle as well as 6 days after the injection of CTX (Fig. 3c). In line with our previous report using pharmacological inhibition of miR-501 [32], we observed that miR-501^ΔMP^ mice form smaller myofibers than control mice following adult skeletal muscle regeneration (Fig. 3d). Bulk RNAseq analysis of the regenerating muscles identified 89 genes significantly downregulated and 146 genes significantly upregulated in miR-501^ΔMP^ mice compared to control mice (p<0.05) (Fig. 3e). Intriguingly, immunoglobulin heavy constant alpha (*Igha*), immunoglobulin kappa light chain (*Igkc*) and joining chain of multimeric IgA and IgM (*Jchain*) were part of the top regulated genes and the term “immunoglobulin receptor binding” was significantly enriched in gene ontology (GO) analysis for molecular function (Fig. 3e). Increased gene expression of this cluster was confirmed using qRT-PCR in a separate cohort (Fig. 3f). Immunofluorescence analysis for IgA in the regenerating muscle of miR-501^ΔMP^ mice revealed a significant increase in the number of IgA^+^ myofibers with CD138^+^ activated plasma cells in their vicinity compared to control littermates (Fig. 3g, Fig. S5a), which is indicative of myofiber necrosis [41]. We observed enhanced myofiber necrosis as early as 2 days after CTX-injection (Fig. 4a), which resolved completely after the completion of muscle regeneration (day 30, Fig. S5b). CTX incubation of myotubes generated from miR-501^ΔMP^ mice *in vitro* replicated the heightened susceptibility of these fibers to damage and significantly enhanced the release of the cell damage marker LDH into the medium (Fig. 4b). Proliferation rate and capacity to form multinucleated myotubes *in vitro* did not differ between miR-501^ΔMP^ and WT myoblasts (Fig. S6a,b). We also did not observe differences in myotube diameter or expression of the muscle differentiation markers *Myog* or *Myh3* (Fig. S6b,c). Therefore, we conclude that the enhanced immunological response detected in the RNAseq dataset results from loss of resilience and increased myofiber damage in the regenerating muscle in the absence of miR-501. To understand which type of insult causes myofiber damage, we subjected naïve miR-501^ΔMP^ mice and control littermates to a single bout of eccentric exercise. Muscles from miR-501^ΔMP^ mice are highly susceptible to stress, since exercise induced significantly more muscle damage in miR-501^ΔMP^ mice as compared to wildtype littermates (Fig. 4c, Table 1). Together, these results demonstrate that miR-501 is required for the size as well as resilience of myofibers *in vivo*. Lack of miR-501 and the presence of the MP^CD74^ cells during muscle formation predisposes newly formed myofibers to myopathy due to smaller myofiber diameter and decreased resilience to injury and exercise.

**Table 1:** **Genetic ablation of miR-501 results in increased muscle fiber damage upon eccentric exercise.** χ2 test of Evan’s Blue Dye (EBD) staining in muscle fibers of Tibialis Abterior (TA) and Gastrocnemius (Gas) 24 h after eccentric exercise; *n* = 4.

		Sections without EBD fibers	Sections with EBD+fibers	% of injured/total fibers	χ2	p - value
TA	Pax7Cre	89.36 % (42±0.22)	10.64 % (5±1.08)	0.112%	19.58	<0.0001
	miR-501ΔMP	46.81 % (22±0.96)	53.19 % (25±2.16)	1.005%		
Gas	Pax7Cre	65.00 % (26±0.71)	35.00 % (14±1.15)	0.186%	10.38	0.0013
	miR-501ΔMP	29.27 % (12±0.54)	70.73 % (29±1.19)	0.518%		

### 3.4 miR-501 controls sarcomeric gene expression in muscle cells from mice and humans

**Figure 5:**
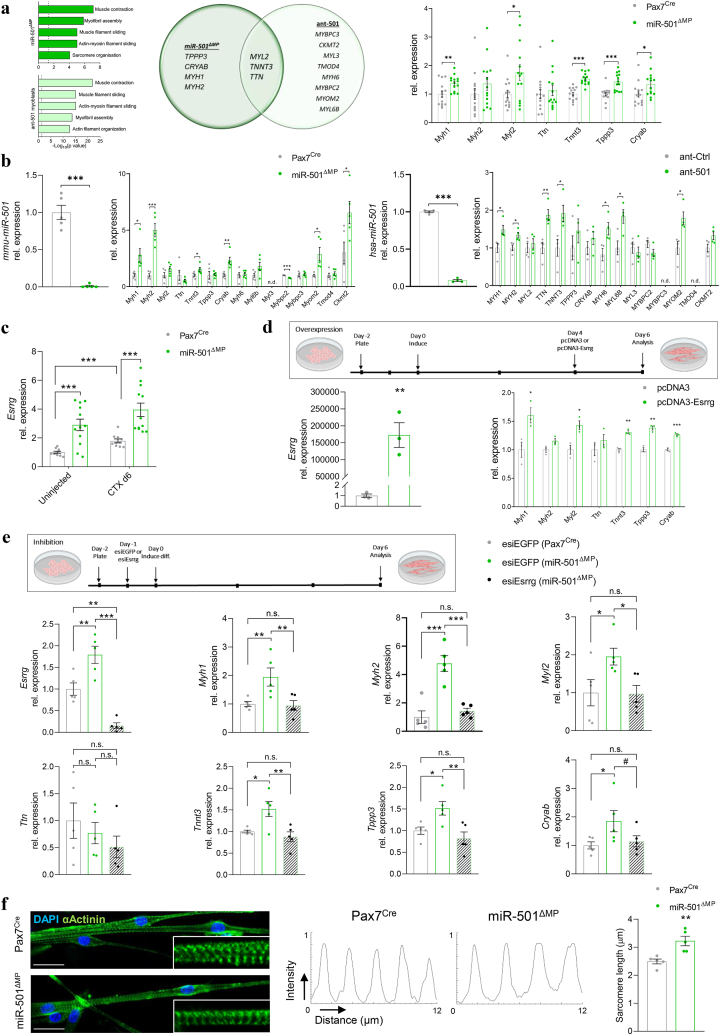
**Changes in sarcomeric gene expression after deletion of miR-501, affecting sarcomere formation. a** Gene ontology (GO) pathway analysis for biological process in TA muscles at day 6 (dark) and in primary myoblasts transfected with control antagomir or antagomir-501 and harvested after 48 h (light), the overlap between the genes shown as Venn diagram, and expression of sarcomeric genes in TA at day 6; *n* = 14. **b** Expression of mmu-miR-501 and sarcomeric genes in mouse primary myotubes at day 6 of differentiation; *n* = 5 independent cell cultures (left), and hsa-miR-501 and sarcomeric genes in human primary myotubes transfected with control antagomir (ant-Ctrl) or antagomir-501 (ant-501) and differentiated for 6 days; *n* = 4 independent cell cultures (right). **c** Expression of Esrrg in TA muscles at baseline or 6 days post-CTX induced regeneration; *n* = 10 Pax7^Cre^ vs. 12 miR-501^ΔMP^. **d** Overexpression of Esrrg using pcDNA3 plasmid or empty vector and effect on sarcomeric genes in wild type mouse primary myotubes; *n* = 3. **e** RNAi of *Esrrg* in mouse primary myotubes and resulting effect on *Myh1*, *Myh2*, *Myl2*, *Ttn*, *Tnnt3*, *Tppp3*, and *Cryab*; *n* = 5 independent cell cultures. **f** miR-501^ΔMP^ and control myotubes were stained using anti-αActinin, and DAPI immunofluorescence and quantified for their sarcomere length using the distance between the peaks in staining intensity. Experiments were performed on 12-week-old male mice and all data is shown as mean ± SEM; qPCR data was normalized to snoRNA234 (mmu-miR-501), U6 small nuclear RNA (hsa-miR-501) or 18S ribosomal RNA. f shows representative images and represents the analysis of at least 150 sarcomeres in 3 different fields of view per cell culture; Scale bar = 50 μm. Significance was evaluated by t-test (a, b, d, f), one-way (e), and two-way analysis of variance (ANOVA; c); ∗: p < 0.05, ∗∗: p < 0.01, ∗∗∗: p < 0.001, #: p = 0.07.

To obtain additional insights into the cell intrinsic role of miR-501, we performed gene ontology pathway analysis in our dataset from miR-501^ΔMP^ mice at day 6 after CTX-injection and on our previously reported dataset from myoblasts treated with antagomir-501 [32]. Strikingly, “muscle contraction” was the top 1 enriched cluster after miR-501 depletion *in vivo* as well as *in vitro* (Fig. 5a). The next top 5 clusters were also related to sarcomere and actin filament organisation. 3 of the 7 significantly upregulated genes encoding structural proteins of the sarcomere were concurrently upregulated in regenerating muscle lacking miR-501 and antagomir-501 treated myoblasts (Fig. 5a). The sarcomere gene signature was confirmed using qRT-PCR in miR-501^ΔMP^ mice *in vivo* (Fig. 5a) as well as *in vitro* in differentiated myotubes obtained from miR-501^ΔMP^ mice (Fig. 5b). Importantly, the effect of miR-501 on sarcomeric genes was conserved in humans. Human primary myotubes differentiated in the presence of antagomir-501 also displayed upregulation of sarcomeric genes compared to control (Fig. 5b). In order to identify the relevant target of miR-501 that causes the sarcomeric gene regulation we turned to our previously validated list of six genes that were selected based on mRNA sequencing in primary myoblasts transfected with either control antagomir or antagomir-501 [32]. Strikingly, this list included estrogen-related receptor gamma (*Esrrg*), a constitutively active nuclear hormone receptor that is important for contractile function in skeletal muscle [42]. Consistent with being a target gene of miR-501, *Esrrg* expression was upregulated in mice lacking miR-501 in both uninjected muscle as well as regenerating muscle at day 6 after CTX injection (Fig.5c). In addition, overexpression of Esrrg during myotube differentiation mirrored the induction of sarcomeric gene expression observed in myotubes lacking miR-501 (Fig 5d). Importantly, myotubes lacking miR-501 also revealed induced levels of *Esrrg* and upregulation of sarcomeric gene expression in these cells was prevented by depletion of *Essrg* using esiRNA (Fig.5e). Together, these findings identify *Esrrg* as the target gene of miR-501 that is responsible for the effect of miR-501 on sarcomeric gene expression. Based on these changes, we hypothesized that miR-501 is required for the assembly of the sarcomere. Indeed, using immunofluorescence for the sarcomere protein alpha-actinin (a Z-line component [43]) we detected a significantly increase in sarcomere length in miR-501^ΔMP^ myotubes compared to control (Fig. 5f).

Next, we were interested to find out how the top 1 GO pathway cluster “muscle contraction” is represented in the single cell sequencing data from miR-501^ΔMP^ mice and controls, and if sarcomere formation is dependent on stem cell heterogeneity. Heatmap analysis revealed a similar pattern in gene expression between committed MPs from miR-501^ΔMP^ and control mice (Fig. S7). In contrast, the muscle contraction cluster markedly differed between the MP^CD74^ cells and the committed myogenic progenitor populations from both groups. Thus, the myofibers arising from the MP^CD74^ cells are likely already predisposed to abnormalities in sarcomere length. We conclude that miR-501 determines a novel myogenic progenitor lineage (MP^CD74^) that largely differs in gene expression relating to muscle contraction compared to canonical committed MPs and which confers physiological perturbations on myofibers once muscle differentiation is completed.

### 3.5 MP^CD74^ is upregulated in aged skeletal muscle where miR-501 is downregulated

**Figure 6:**
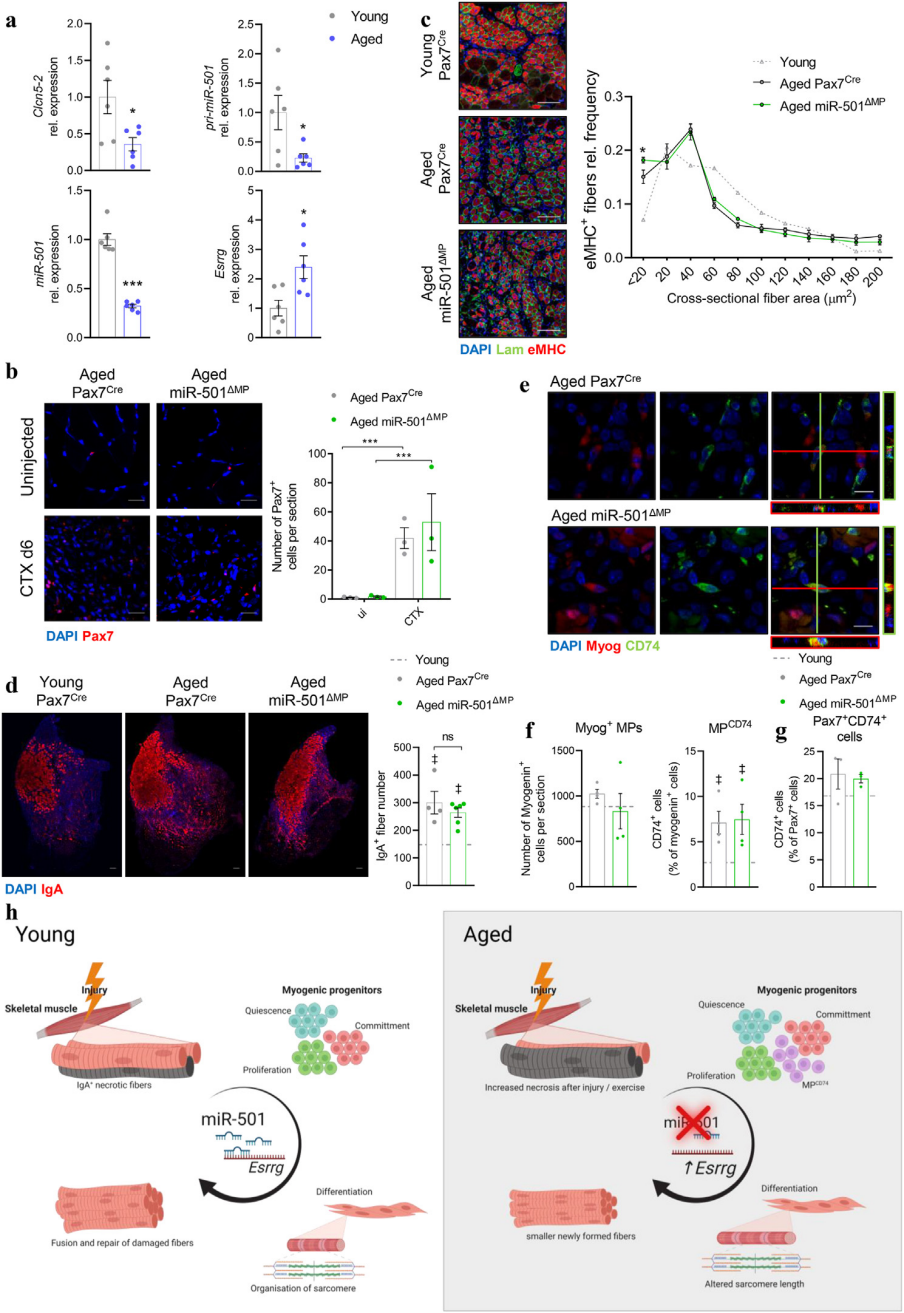
**Aging muscle is characterised by decreased miR-501 levels and presence of MP**^**CD74**^**cells. a** Expression of *Clcn5-2*, pri-miR-501, miR-501-3p, and *Esrrg* in aged mice (22 months) compared to young control (3 months); *n* = 6. **b-d** 24-month-old Pax7^Cre^ and miR-501^ΔMP^ were subjected to CTX-induced muscle regeneration and assessed at day 6. **b** Muscle cross sections of uninjected or regenerating TA muscle were stained using anti-Pax7 and DAPI immunofluorescence. Sections were quantified based on Pax7^+^ cells per section; *n* = 3. **c** Muscle sections were stained using anti-Laminin, anti-eMHC, and DAPI immunofluorescence and quantified compared to young Pax7^Cre^ control mice (3 months); *n* = 5 Pax7^Cre^ vs. 6 miR-501^ΔMP^. Mean of young mice is shown as dashed grey line. **d** Cross sections were stained using anti-IgA and DAPI immunofluorescence and quantified for IgA^+^ fiber number; *n* = 4 Pax7^Cre^ vs. 6 miR-501^ΔMP^. **e** MP^CD74^ in aged mice as shown using myogenin and CD74 co-staining **f** Quantification of Myogenin^+^ cells and MP^CD74^ (Myogenin^+^CD74^+^) cells per section at day 6 following CTX-induced muscle injury; n = 4. **g** Quantification of Pax7^+^CD74^+^ per section at day 6 following CTX-induced muscle injury; n = 3. **h** Model for the role of miR-501 in regeneration of young and aged skeletal muscle. Experiments were performed on male mice and all data is shown as mean ± SEM; qPCR data was normalized to sno-234 (miR-501) or 18S ribosomal RNA. For comparisons with young mice, additional images were taken from cohorts shown in figure 3d (eMHC), and 3g (IgA); all images are representative. Quantifications were performed on sections from two different heights within the muscle per mouse; Scale bar = 20 μm (b), 100 μm (c, d), 10 μm (f). Mean of young mice is shown as dashed grey line; ‡: significance vs. young. Significance was evaluated by t-test (a) and one-way (b, d, f), and two-way analysis of variance (ANOVA; c); ‡: p < 0.01, ∗: p < 0.05, ∗∗∗: p < 0.001.

The smaller muscle fibers of skeletal muscle of miR-501 knockout mice are reminiscent of the small diameter myofibers in aged skeletal muscle [[44], [45]]. We therefore investigated the expression of miR-501 in skeletal muscle from aged and young mice, and assessed muscle regeneration in a cohort of aged miR-501^ΔMP^ mice and their aged littermates. miR-501 expression as well as levels of its host gene *Clcn5-2* and the pri-miR-501 were markedly downregulated in wildtype aged versus young skeletal muscle while expression of its target gene *Esrrg* was upregulated (Fig. 6a). Moreover, CTX-induced muscle regeneration in aged mice mirrored the phenotype of miR-501^ΔMP^ mice. While the number of Pax7^+^ MuSCs was not affected by the loss of miR-501 (Fig. 6b), aged mice formed significantly smaller neofibers (Fig. 6c) and displayed enhanced formation of IgA^+^ necrotic fibers compared to younger animals (Fig. 6d). Intriguingly, deletion of the miR-501 gene did not further aggravate these deficiencies, aside from a minor increase in the frequency of < 20 μm^2^ fibers (Fig. 6c). These results indicate that miR-501 is already inactive during muscle regeneration in aged skeletal muscle and that deletion of miR-501 does not further exacerbate the defects in muscle regeneration. Indeed, the number of MP^CD74^ was similar in both aged miR-501^ΔMP^ mice and their aged control littermates and higher as compared to young mice (Fig. 6e,f). There was no significant difference for Pax7^+^/CD74^+^ cells between aged wildtype and knockout mice or aged and young mice (Fig.6g). Together, these data demonstrate that consequential deficiencies in muscle regeneration in aged skeletal muscle can be attributed to miR-501 and the MP^CD74^ cells that depend on miR-501 expression.

## 4 Discussion

Our work reveals a novel group of myogenic progenitor cells, MP^CD74^ that is under control of miR-501 during skeletal muscle regeneration, and that is upregulated in aged skeletal muscle where miR-501 is downregulated (Fig.6h). MP^CD74+^-aged muscle mirrors the phenotype of miR-501 knockout mice, characterized by decreased fiber size and increased fiber necrosis after injury. The reduction in myofiber size during aging has been mainly attributed to the depletion of MPs [[14], [15], [16], [17]], although induced lifelong depletion of MPs at 4 months of age did not affect myofiber size in sedentary mice [46]. Constitutive or tissue-specific deletion of miR-501 during embryonic development reduced myofiber size in adult skeletal muscle even during homeostasis. Our data therefore suggest that perhaps it is the intrinsic molecular characteristics of the various MuSC subpopulations, rather than the pool size of MuSCs, which affects the aged muscle.

Heterogeneity in MuSCs during regeneration of aged muscle is a novel finding, but global differences between aged muscle stem cells and young controls have been previously described. Whilst aged muscle stem cells are still able to maintain a high clonal diversity, at least in the absence of repeated bouts of injury [47], gene and protein expression shows greater variability compared to cells from young muscle, which has been related to DNA methylation and epigenetic drift [48]. Key alterations were described in extracellular matrix-related genes, which might explain defects in the aged stem cell niche [48]. Interestingly, a previous study using single nuclei sequencing of adult myofibers revealed 3 novel myonuclear subpopulations in aged compared to young skeletal muscle [49], indicating that heterogeneity could also be relevant in myonuclei during aging. Therefore, heterogeneity along the developmental trajectory of MuSCs might in the future prove to be a crucial determinant for disturbed muscle regeneration in the aged state.

Sampling rare cell types is a limitation of single cell RNAseq. While *CD74* positive MuSCs are present in public single cell RNAseq data sets from adult mice and healthy human skeletal muscle (Fig. S9) [[50], [51]], their frequency is too low to be classified as a separate MuSC subpopulation. Furthermore, datasets for single-cell sequencing from aged human skeletal muscle, where the MP^CD74^ cells should appear, are not available to date. An in depth characterization of MP^CD74^ cells will be crucial to understand the function of these cells in human skeletal muscle in the future. Interestingly, single RNAseq from human skeletal muscle shows that *CLCN5*, the host gene of miR-501, is specifically expressed in MPs (Fig. S9) [51], indicating that miR-501 could also impact MuSC heterogeneity in humans since both host and miRNA are co-expressed [32]. We were successful to detect the MP^CD74^ cells by using a microRNA knockout model, where this subpopulation was 21 % of total MuSCs, highlighting again that conferring robustness to developmental decisions is a key feature of the miRNA family [52]. Our data shows that deletion of stem-cell specific miRNAs combined with single cell sequencing approaches is a powerful strategy to uncover novel stem cell populations in aged tissues. The MP^CD74^ cells revealed the characteristic gene expression signature of differentiating myogenic progenitors including the fusogenic genes myomaker and myomixer. Gene expression therefore indicates that they participate in the formation of newly formed fibers during muscle regeneration. A definite prove of this assumption will, however, require the generation of transgenic mice that allow for lineage tracing studies. The fact that the differentiation of myoblasts lacking miR-501 *ex vivo* did not alter myotube size supports the notion that the MP^CD74^ cells are involved in the observed changes of myofiber size *in vivo*. To test the causal role of MP^CD74^ cells for the formation of myofibers will need further testing in cell transplantation assays. Lastly, while the smaller myofiber diameter in miR-501 knockout mice are reminiscent of aged mice, mice lacking miR-501 also showed an increased number of myofibers, a phenomenon which is not found in aged skeletal muscle. Aging might provide an adverse environment that prevents the compensatory increase in myofiber number observed in miR-501 knockout mice.

Activation of the immune system is a major pathway in the transcriptomic analysis of miR-501 knockout mice. Genes encoding for components of immunoglobulins were the highest upregulated group of genes during muscle regeneration in the absence of miR-501. The increased number of activated CD138^+^ plasma cells adjacent to necrotic IgA^+^ fibers provides a plausible explanation for this gene signature and has been described for inflammatory myopathies in humans [[53], [54]]. We therefore conclude that the enhanced activation of the immune system is the consequence of increased myofiber necrosis in miR-501 knockout mice. Our data shows for the first time that damage-induced regeneration in aged muscle is also characterized by an increased frequency of IgA^+^ myofibers. Decreased myofiber resilience might contribute to the impaired capacity of aged muscle to regenerate, and modulation of plasma cell activation could offer novel means to improve muscle regeneration during aging.

An inflammatory gene signature mainly characterized by expression of the *Cd74* gene is further observed in the novel cell population that we describe during muscle regeneration. *Cd74* encodes a transmembrane protein that is expressed on antigen-presenting cells and functions as a major histocompatibility complex II chaperone [55]. This inflammatory signature might also be the consequence of the enhanced myositis similar to the observed activation of plasma cells. Indeed, *Cd74* expression was absent in *ex vivo* myoblasts and not induced when miR-501 was inhibited using antagomirs (RNA sequencing data [32]: FPKM <5 for *Cd74* in all conditions). Regardless of the cause of CD74 activation in miR-501 knockout mice and aged muscle stem cells, our data strongly support previous observations that activation of the immune system characterizes aged skeletal muscle. Myonuclear subpopulations in aged skeletal muscle from mice [49] and muscle biopsies from aged humans [56] have revealed enrichment of genes associated with the immune response. Moreover, a single-cell transcriptomic atlas identified the inflammatory environment as a common hallmark of aging with *Cd74* as a top candidate gene in aged macrophages within spleen and liver [57]. Our results extend these finding to MuSCs in aged muscle.

Regulation of the sarcomere was the second major pathway affected by the loss of miR-501. Genes encoding structural proteins within the sarcomere represented the highest induced biological process ontology during muscle regeneration in miR-501 knockout mice and antagomir-501 treated myoblasts. We confirmed the sarcomeric gene signature in myoblasts from miR-501 knockout mice and in antagomir-501 treated human primary myoblasts, illustrating that this effect of miR-501 is both cell intrinsic and conserved between mice and humans. The search for predicted miR-501 targets identified estrogen-related receptor gamma (*Esrrg*) as the mediator of sarcomeric gene regulation downstream of miR-501. The importance of Esrrg for skeletal muscle contractility and metabolism has been previously demonstrated using knockout myocytes [58] or overexpression in transgenic mice [[59], [60]]. Interestingly, in these models *Esrrg* is not only essential for myotube formation and the contractile fiber part, but also promotes a switch towards more oxidative fibers. In our miR-501 knockout mice fiber typing remained unchanged. These differences could be explained by the degree of regulation of *Esrrg* in miR-501 knockout mice compared to transgenic mice as well as by the time point of *Esrrg* regulation during muscle regeneration. miR-501 is already activated in myogenic progenitors, while the transgenic overexpression of *Esrrg* was achieved in mature myofibers under the alpha-skeletal actin promoter [60] or the muscle creatine kinase promoter [59]. Importantly, the induction of *Esrrg* expression in skeletal muscle of miR-501 knockout mice resembles the extend of induction of *Esrrg* observed after exercise [59]. Therefore, miR-501 knockout mice present a unique model to explore the fine-tuning of *Esrrg* expression during muscle regeneration and its therapeutic value for improving sarcomere formation in aged muscle.

Binenbaum et al [61] and Zhou et al [62] showed that miR-501-3p is enriched in exosomes derived from M2 macrophages compared to M1 macrophages. Since we did not observe differences between global miR-501 KO mice and tissue-specific deletion of miR-501 in Pax7^+^ cells our data provide no evidence that exosomal transfer of miR-501-3p is required for muscle regeneration under normal conditions. However, miR-501-3p transfer from M2 macrophages to intact MPs remains an interesting possibility in disease states that we did not address with our global KO mouse.

The consequences of sarcomeric gene regulation during myotube formation were striking. miR-501 knockout myotubes revealed elongated sarcomeres which characterizes immature sarcomeres at early stages of myotube formation [63]. The structure of the sarcomere plays a critical role in conferring resilience to exercise-induced muscle damage [64] and determining myofiber size [65]. The disruption of sarcomeric gene expression and increased sarcomere length in the absence of miR-501 therefore provide a plausible cause for the decrease in muscle fiber resilience to injury and exercise and the smaller myofiber size. The importance of appropriate synthesis, folding, and incorporation of sarcomeric components is highlighted by the frequent dysregulation of sarcomeric proteins in diseases affecting striated muscle [66] as well as in aged human skeletal muscle [67].

## 5 Conclusions

In conclusion, our study identifies a regulatory mechanism through miRNA activation in MuSCs, which directly influences the quality of adult skeletal muscle. Targeting stem cell heterogeneity in aged skeletal muscle represents a novel therapeutic strategy to restore muscle regeneration during aging. We propose that depletion of MP^CD74^ could rescue myofiber diameter and the resilience of aged skeletal muscle to injury and exercise.

## Author contributions

A.F. designed, performed, and analyzed experiments and wrote the manuscript, E.L. participated in immunofluorescence analysis and flow cytometry. J.K. supervised the study, designed, and analyzed experiments and wrote the manuscript. All authors discussed the results and commented on the manuscript.

## Data Availability

The authors declare that all data supporting the findings of this study are available within the article and its supplemental information files or from the corresponding author upon reasonable request. The RNAseq data described in this report have been deposited in GEO database (GSE199111 for RNA seq in TA and GSE200501 for sc-RNA seq).
