# Supplementary material for: microRNA-501 controls myogenin+/CD74+ myogenic progenitor cells during muscle regeneration
Source: Mol Metab. 2023 Mar 11;71:101704. doi: 10.1016/j.molmet.2023.101704 (PMC10033748; doi:10.1016/j.molmet.2023.101704)
Supplement: Multimedia component 1 [file mmc1.pdf]

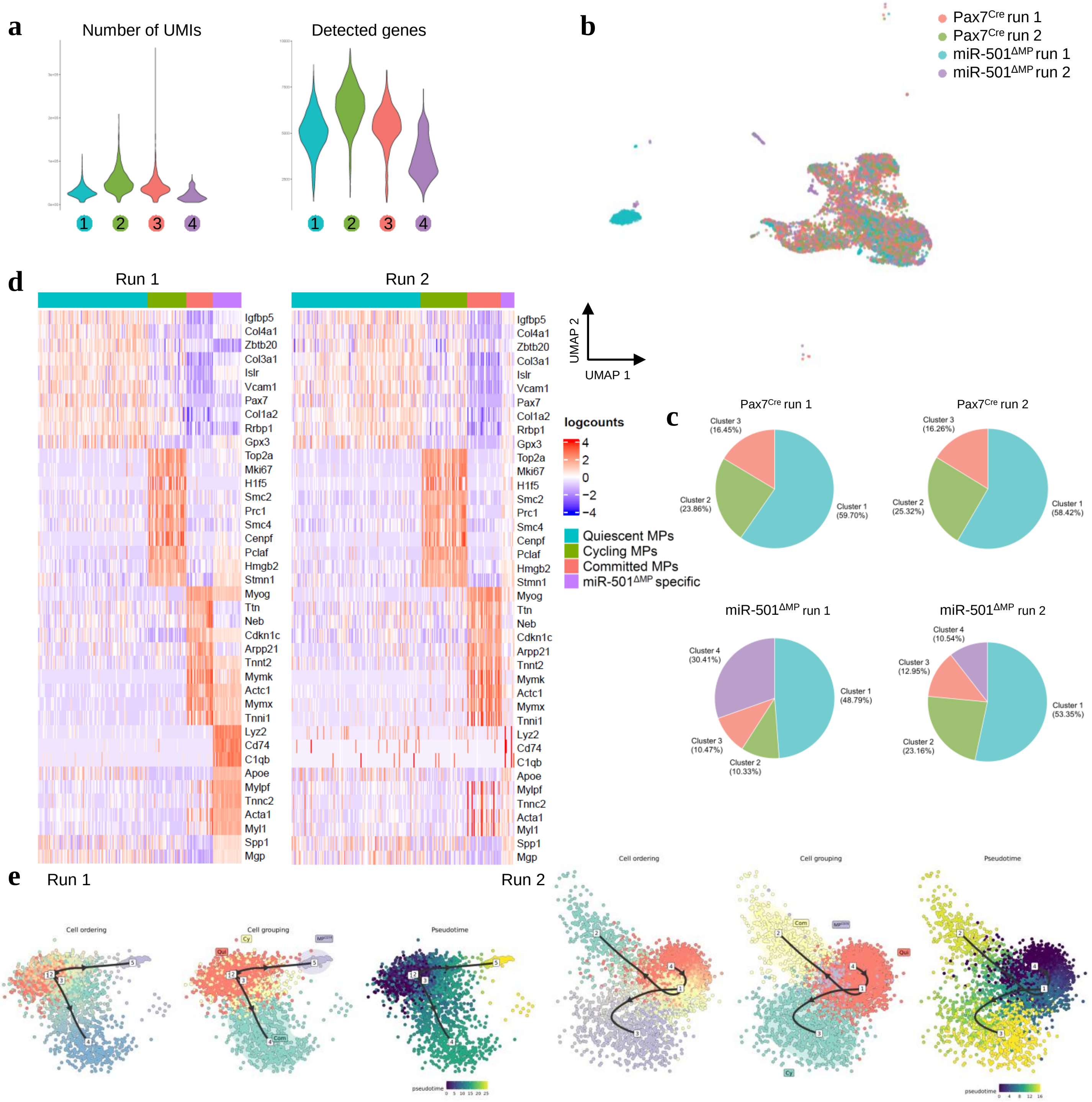

Figure S1: MP^{CD74} show distinct gene expression pattern compared to committed MPs. a Violin plot for number of UMI and detected genes for each cluster. **b** UMAP of single-cell RNA sequencing of samples merged from two independent runs. **c** Cluster composition of each sample divided into Pax7^{Cre} (2578 cells in run 1; 1544 cells in run 2) and miR-501$^{\Delta MP}$ (2111 cells in run 1; 1822 cells in run 2). **d** Heatmap of top 10 marker genes per cluster shown separate for run 1 (left) and run 2 (right) for quiescent, cycling, and committed MPs, and MP^{CD74}. **e** Slingshot analysis of run 1 (left) and 2 (right).

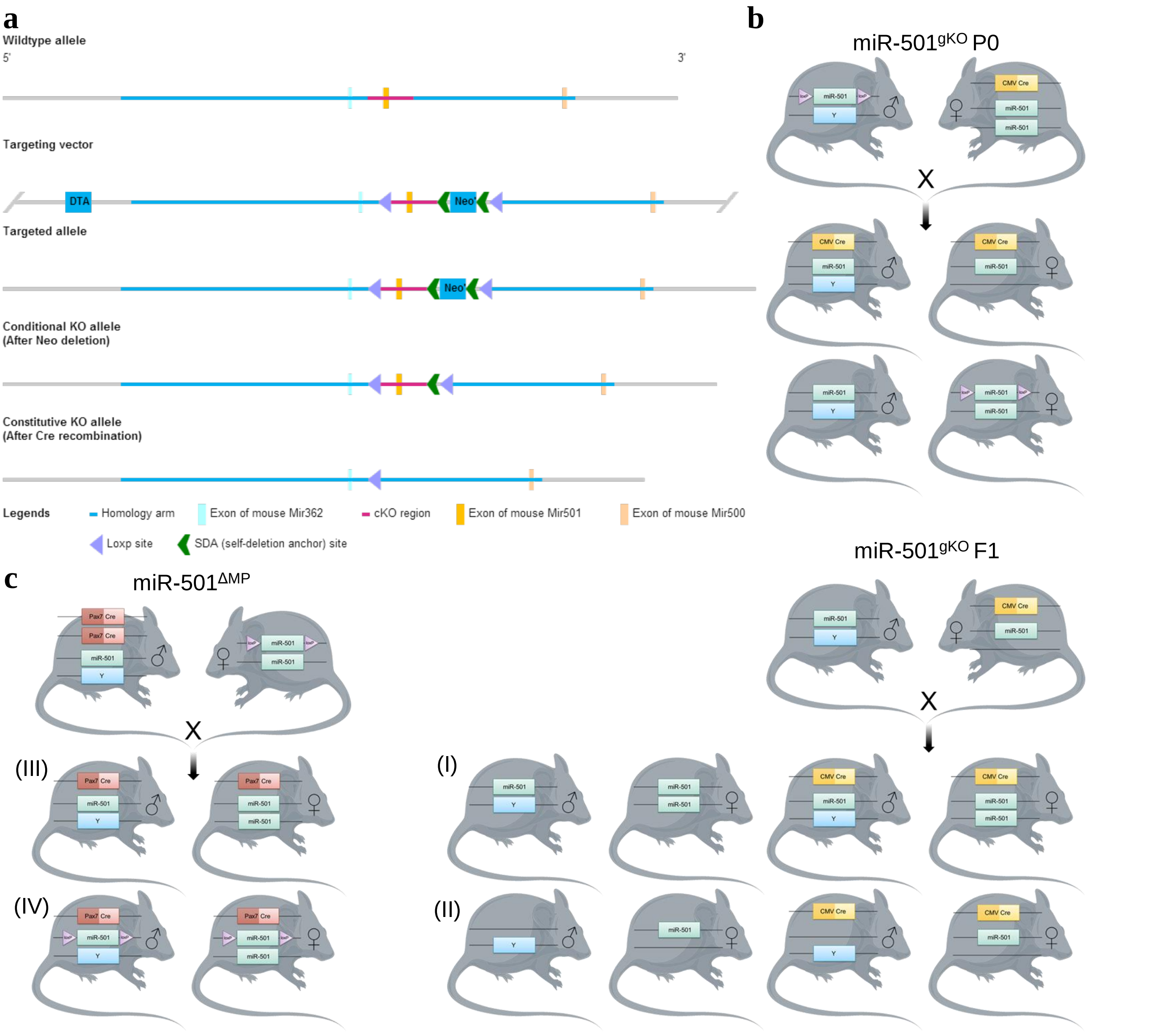

Figure S2: Generation of miR-501 knockout mouse models. a Targeting Strategy for the generation of miR-501^{flox} mice. **b** Breeding of P0 and F1 generations of miR-501^{gKO} mice (II) and wt littermates (I). **c** Generation of miR-501$^{\Delta MP}$ (Pax7^{Cre}miR-501$^{fl/Y}$; IV) mice and Pax7^{Cre} (III) littermates.

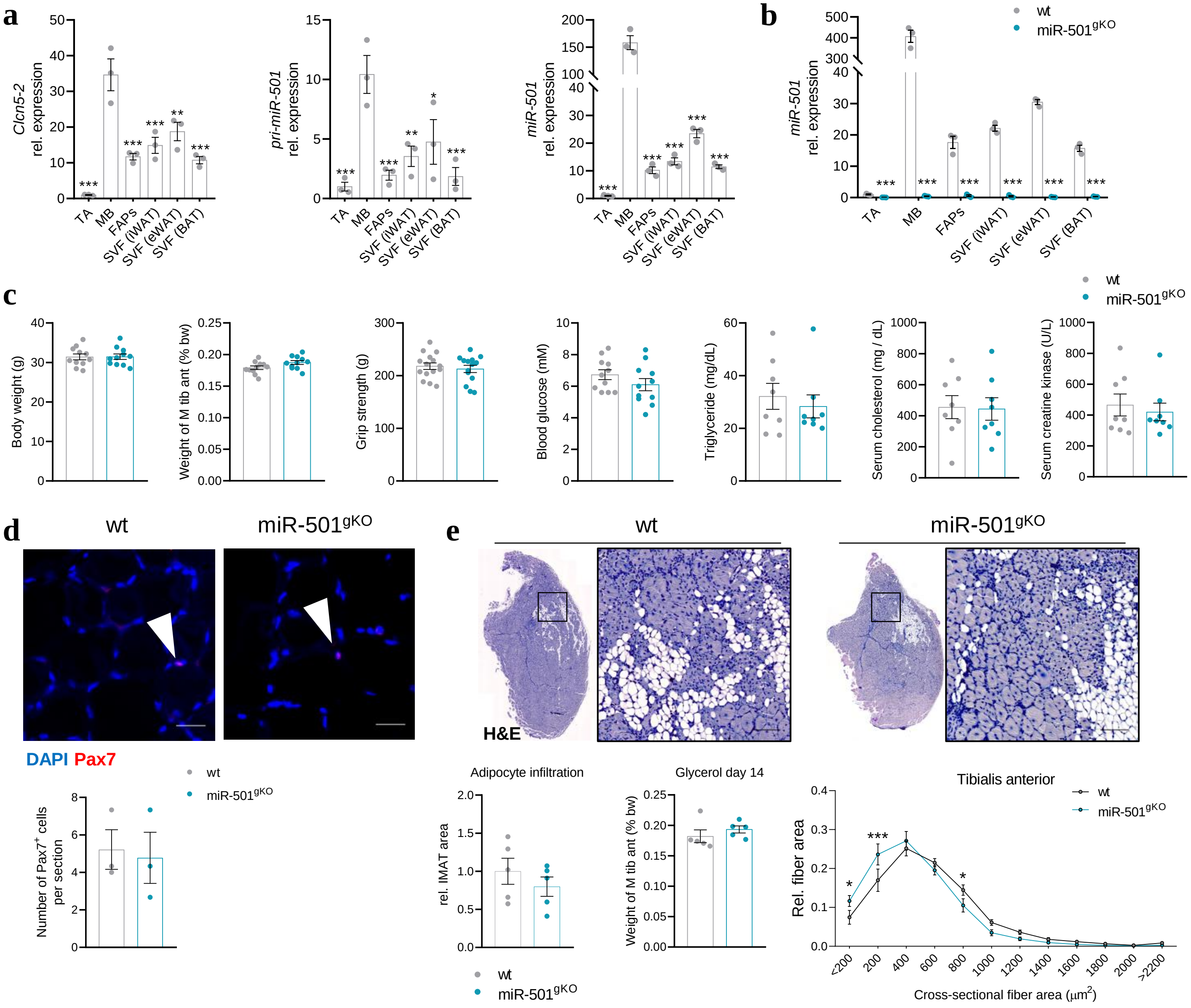

Figure S3: miR-501 is not involved in the emergence of intramuscular adipose tissue (IMAT). a Expression of *Clcn5-2*, pri-miR-501, and miR-501 in tibialis anterior (TA) muscle compared to myoblasts (MBs), fibro/adipogenic progenitors (FAPs), stromal vascular fraction (SVF) of inguinal white adipose tissue (iWAT), epididymal white adipose tissue (eWAT), and brown adipose tissue (BAT) isolated from C57/B6 mice and cultured for 48 h before RNA extraction; *n* = 3. **b** miR-501 expression in TA compared to precursor cell populations isolated from wt and miR-501^{gKO} mice and cultured for 48 h before RNA extraction to confirm miR-501 knockout; *n* = 3. **c** Phenotypical characterization of miR-501^{gKO} mice. Body weight and weight of TA muscle as percent of total body weigh; *n* = 11; Grip strength measurement; *n* = 15 wt *vs.* 14 miR-501^{gKO}; Blood glucose after 5 h of fasting (*n* = 11), and serum triacylglycerides, total serum cholesterol, and serum creatine kinase (CK) in miR-501^{gKO}; *n* = 8. **d** Muscle cross sections of tibialis anterior muscle were stained using anti-Pax7 and DAPI immunofluorescence. Sections were quantified based on Pax7$^+$ cells per section; *n* = 3. **e** Intramuscular adipose tissue (IMAT) formation in miR-501^{gKO} mice 14 days after glycerol injection assessed in muscle cross-sections stained using hematoxylin and eosin (H&E), and weight of TA muscle; *n* = 5. Experiments were performed on 12-week-old male mice and all data is shown as mean ± SEM; qPCR data was normalized to snoRNA234 (mmu-miR-501) or 18S ribosomal RNA. d and e show representative images and the analysis of two consecutive sections at two different heights within the muscle; Scale bar = 20µm (d); 100 µm (e). Significance was evaluated by one-way (a) and two-way (b) analysis of variance (ANOVA) vs. MBs; *$p \leq 0.05$; **$p \leq 0.01$; ***$p \leq 0.001$.

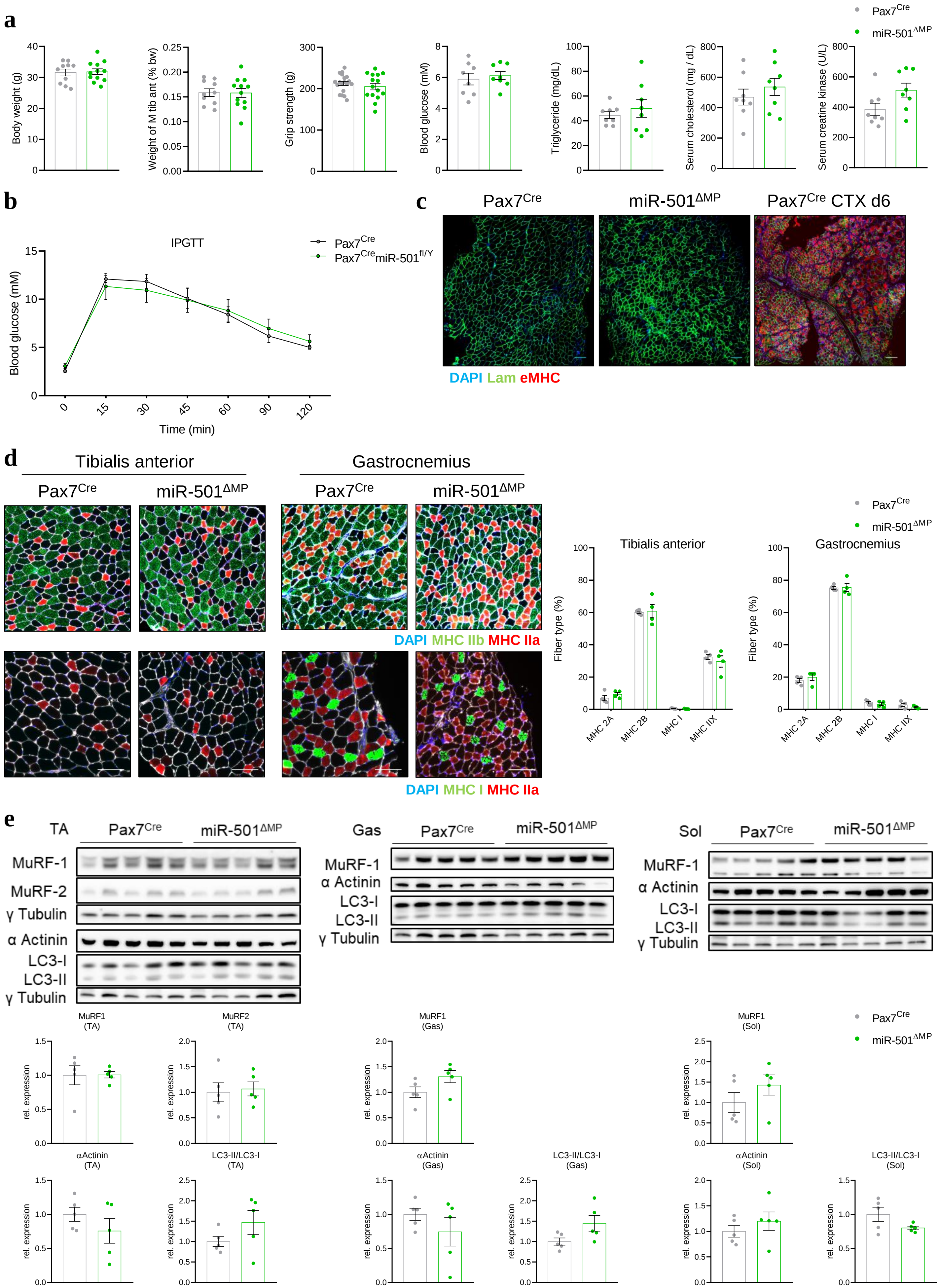

a
Pax7Cre
miR-501ΔMP
Body weight (g)
Weight of M tib ant (% bw)
Grip strength (g)
Blood glucose (mM)
Triglyceride (mg/dL)
Serum cholesterol (mg / dL)
Serum creatine kinase (U/L)
b
IPGTT
Pax7Cre
Pax7CremiR-501fl/Y
Blood glucose (mM)
Time (min)
c
Pax7Cre
miR-501ΔMP
Pax7Cre CTX d6
DAPI Lam eMHC
d
Tibialis anterior
Gastrocnemius
Pax7Cre
miR-501ΔMP
DAPI MHC IIb MHC IIa
DAPI MHC I MHC IIa
Fiber type (%)
MHC 2A
MHC 2B
MHC I
MHC IIX
e
TA
Gas
Sol
MuRF-1
MuRF-2
γ Tubulin
α Actinin
LC3-I
LC3-II
MuRF1 (TA)
MuRF2 (TA)
MuRF1 (Gas)
MuRF1 (Sol)
αActinin (TA)
LC3-II/LC3-I (TA)
αActinin (Gas)
LC3-II/LC3-I (Gas)
αActinin (Sol)
LC3-II/LC3-I (Sol)
rel. expression

Figure S4: Loss of miR-501 does not affect muscle fiber type and homeostasis. a Phenotypical characterization of miR-501$^{\Delta MP}$ mice. Body weight and weight of TA muscle as percent of total body weight; *n* = 10 *vs* 12; Grip strength measurement; *n* = 19 *vs*. 15; Blood glucose after 5h fasting and serum measurements; n = 8. **b** intraperitoneal glucose tolerance test (IPGTT) assessing blood glucose levels at indicated time points after glucose challenge; *n* = 5 *vs* 6. **c** Cross sections of uninjected muscle were stained using anti-Laminin, anti-eMHC and DAPI immunofluorescence; a section of a Pax7^{Cre} control mouse at d6 of CTX-induced regeneration served as positive control. **d** Muscle cross sections of tibialis anterior and gastrocnemius were stained using anti-MHC I, anti-MHC IIa, anti MHC IIb, anti-Laminin and DAPI immunofluorescence. Sections were quantified based on the distribution of fiber types in TA and gastrocnemius with MHC IIx fibers determined as the percentage of unstained fibers; *n* = 4. **e** Cropped western blot of proteins involved in sarcomeric homeostasis in tibialis anterior (TA), gastrocnemius (Gas) and Soleus (Sol) muscles of Pax7^{Cre} and miR-501$^{\Delta MP}$ mice including the densiometric analysis normalized to γ-tubulin (MuRF1, MuRF2, α-Actinin) or as a ratio of LC3-II to LC3-I; *n* = 5. Experiments were performed on 12-week-old male mice and all data is plotted as mean ± SEM. c, d show representative images; d represents the analysis of two consecutive sections at two different heights within the muscle; Scale bar = 100 µm.

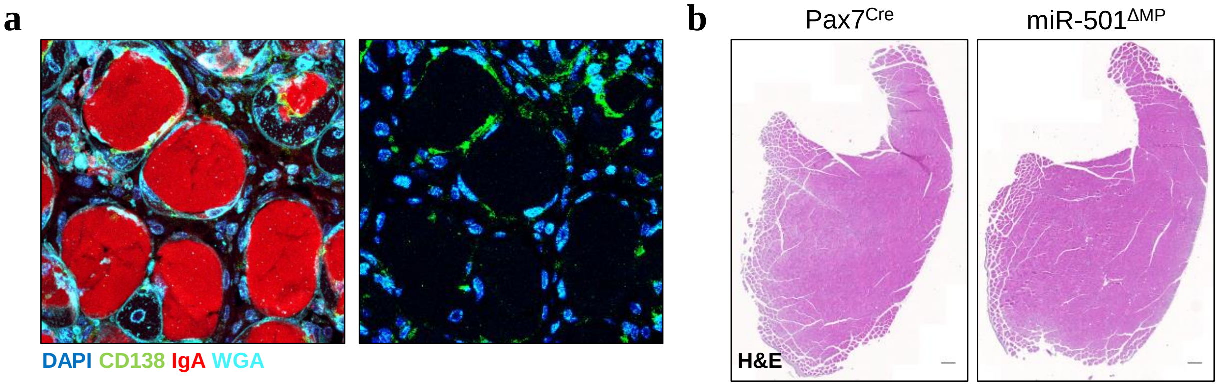

Figure S5: Emergence of CD138^{+} cells around necrotic muscle fibers during muscle regeneration. a Muscle section of Pax7^{Cre} control mouse stained using anti-CD138, anti-IgA, WGA, and DAPI immunofluorescence showing the localization of plasma cells around IgA^{+} muscle fibers. **b** H&E staining of sections at d 30 of CTX-induced muscle regeneration: Scale bar = 200 µm.

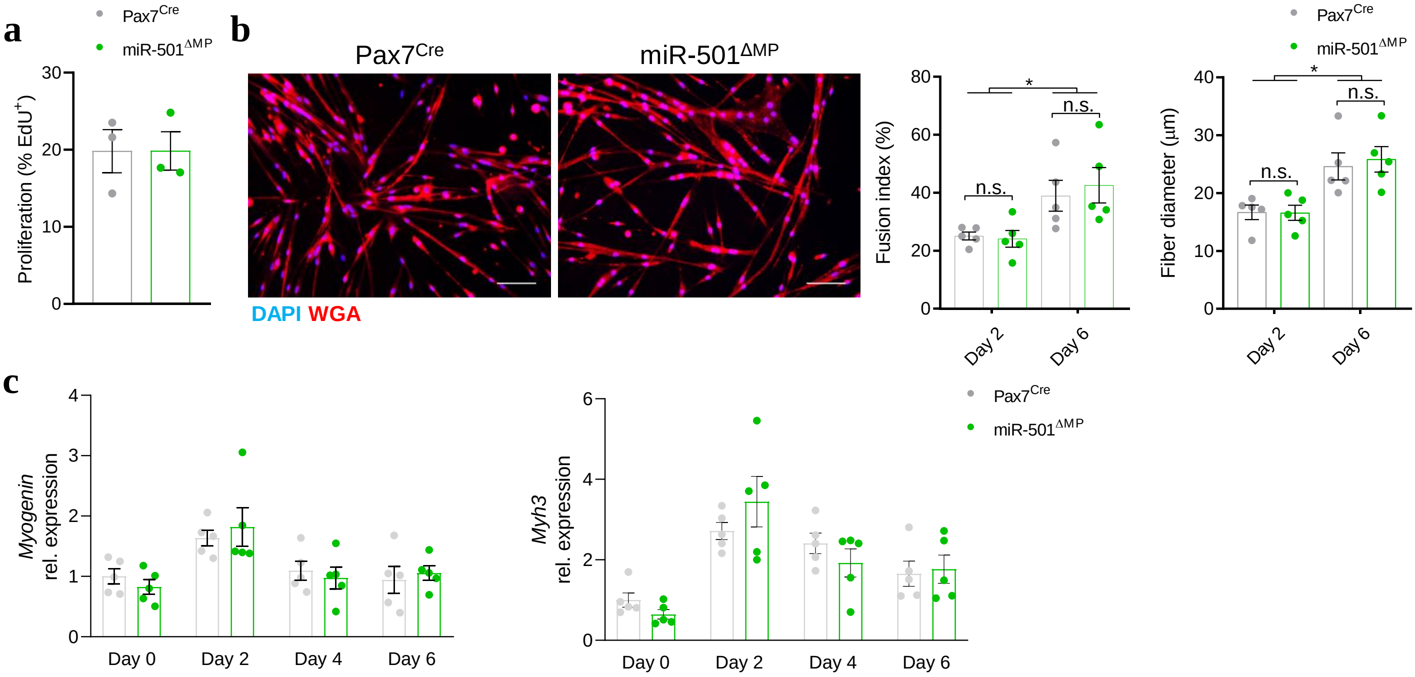

Figure S6: Loss of miR-501 does not impair myoblast proliferation or myotube formation. a miR-501$^{\Delta MP}$ myoblasts isolated from Pax7^{Cre} and miR-501$^{\Delta MP}$ mice and assessed for proliferation rate by EdU incorporation using flow cytometry; n = 3 independent cell cultures. **b** Differentiation of miR-501$^{\Delta MP}$ myoblasts. Myotubes were stained using WGA and DAPI and fusion index (fibers with 3 or more nuclei per total nuclei) as well as fiber diameter was calculated on day 2 and day 6 of differentiation; n = 5 independent cell cultures. c expression of *Myogenin* and *Myh3* at indicated days during differentiation. All data is shown as mean ± SEM; qPCR data was normalized to 18S ribosomal RNA. b shows representative images; Scale bar = 100 µm. Significance was evaluated by one-way analysis of variance (ANOVA); *$p \leq 0.05$.

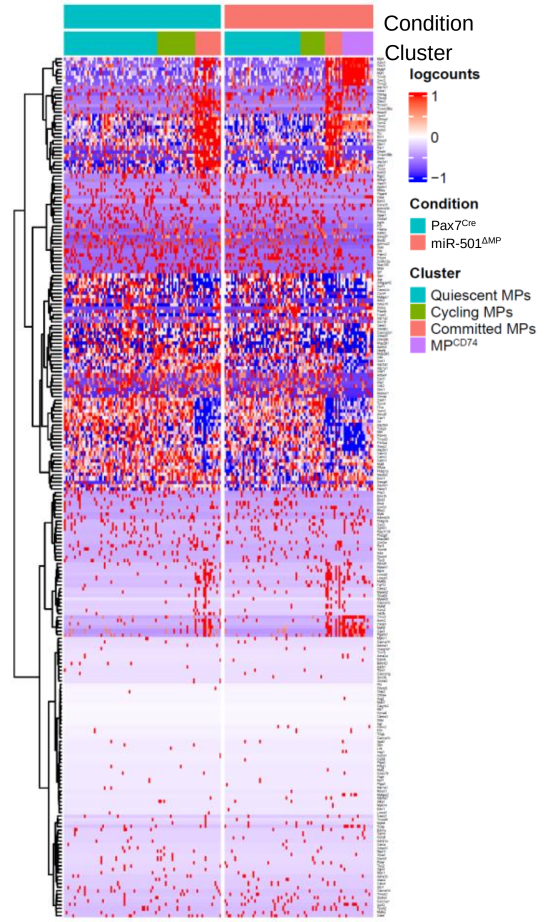

Figure S7: MP^{CD74} differ from committed MPs in sarcomeric gene expression. Heatmap of GO:0006936 (muscle contraction) divided into condition Pax7^{Cre} and miR-501$^{\Delta MP}$ as well as into quiescent, cycling, and committed MPs, and MP^{CD74}.

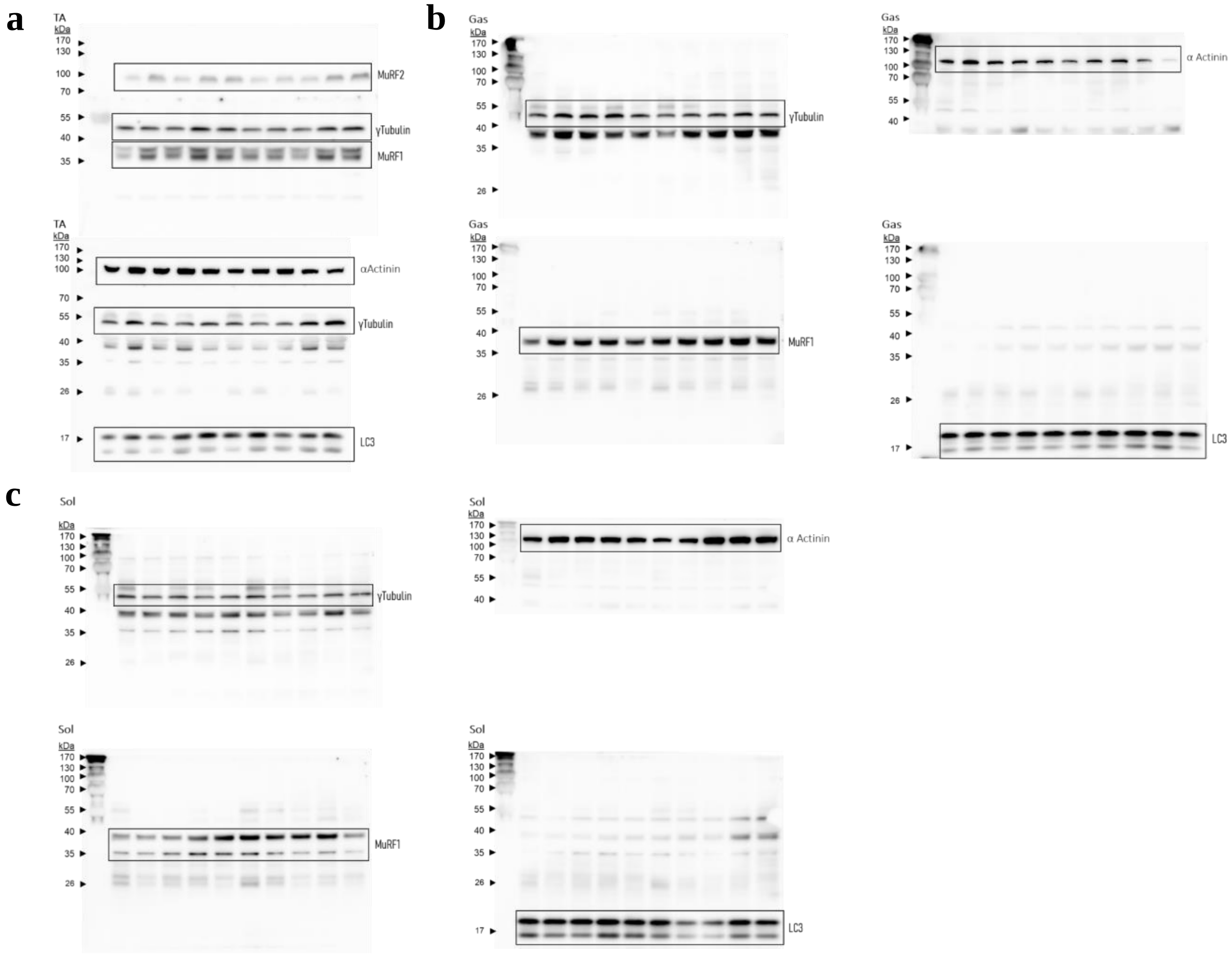

Figure S8: Unedited blots. Full unedited blot of Extended Data Fig. 4e. for Tibialis Anterior (**a**), Gastrocnemius (**b**), and Soleus (**c**).

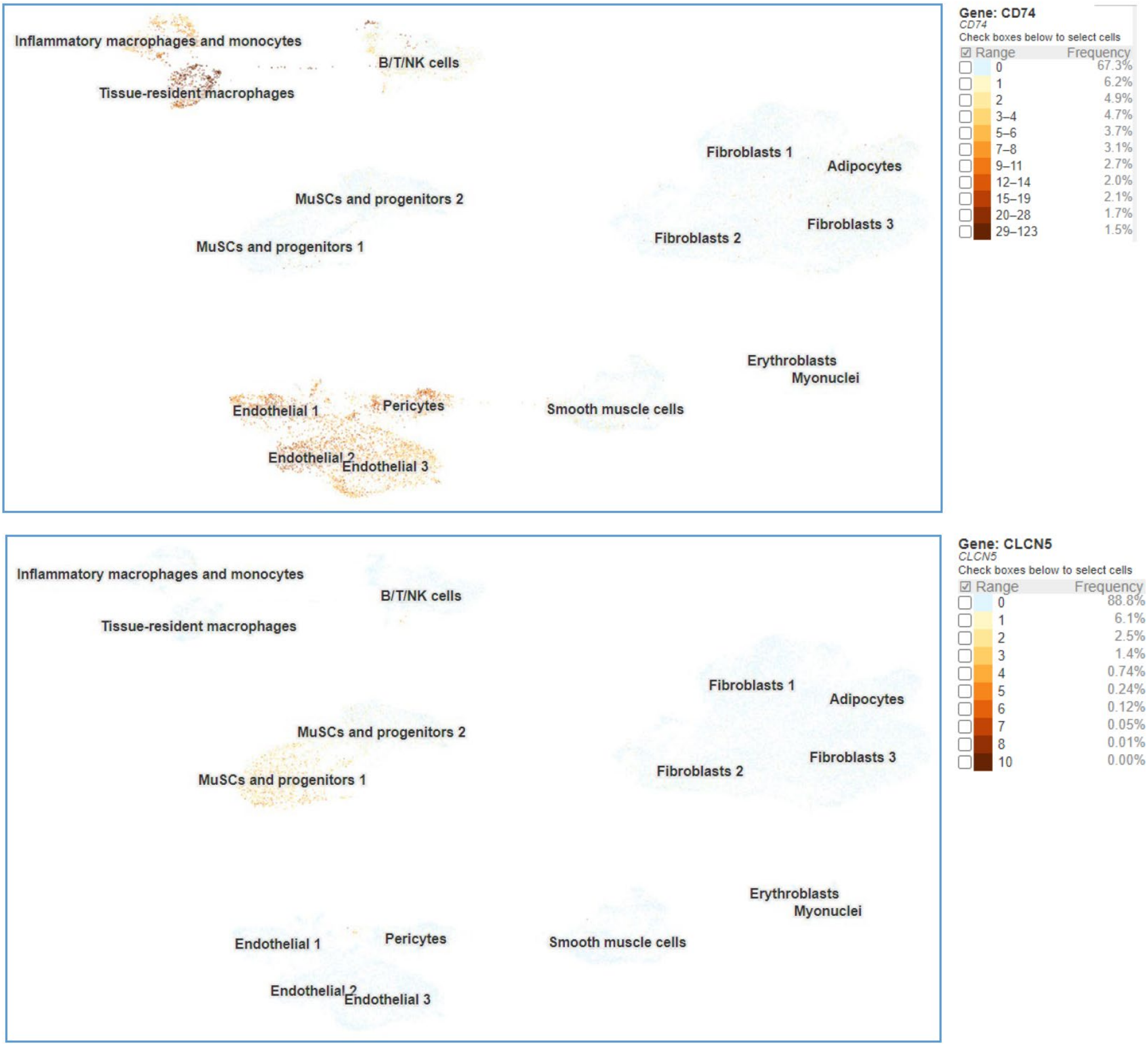

Figure S9: Expression analysis of CD74 and CLCN5 in curated scRNA-seq dataset of human muscle samples from 10 adult donors with diverse anatomical locations. From https://muscle-cell-atlas.cells.ucs.ed [46].
